# Navigating the Purification Process: Maintaining the Integrity of Replication-Competent Enveloped Viruses

**DOI:** 10.3390/vaccines13050444

**Published:** 2025-04-23

**Authors:** Adrian Schimek, Judy King Man Ng, Jürgen Hubbuch

**Affiliations:** 1 ViraTherapeutics GmbH, Bundesstraße 27, 6063 Rum, Austria; 2 Karlsruhe Institute of Technology, Institute of Process Engineering in Life Sciences, Section IV Biomolecular Separation Engineering, Fritz-Haber-Weg 2, 76131 Karlsruhe, Germany

**Keywords:** enveloped virus particles, replication-competent virus particles, virus purification, oncolytic viruses, cancer vaccines

## Abstract

Replication-competent virus particles hold significant therapeutic potential in application as oncolytic viruses or cancer vaccines. Ensuring the viral integrity of these particles is crucial for their infectivity, safety, and efficacy. Enveloped virus particles, in particular, offer large gene insert capacities and customizable target specificity. However, their sensitivity to environmental factors presents challenges in bioprocessing, potentially compromising high quality standards and cost-effective production. This review provides an in-depth analysis of the purification process steps for replication-competent enveloped virus particles, emphasizing the importance of maintaining viral integrity. It evaluates bioprocessing methods from cell culture harvest to final sterile filtration, including centrifugation, chromatographic, and filtration purification techniques. Furthermore, the manuscript delves into formulation and storage strategies necessary to preserve the functional and structural integrity of virus particles, ensuring their long-term stability and therapeutic efficacy. To assess the impact of process steps on particles and determine their quality and integrity, advanced analytical methods are required. This review evaluates commonly used methods for assessing viral integrity, such as infectious titer assays, total virus particle quantification, and structural analysis. By providing a comprehensive overview of the current state of bioprocessing for replication-competent enveloped virus particles, this review aims to guide researchers and industry professionals in developing robust and efficient purification processes. The insights gained from this analysis will contribute to the advancement of virus-based therapeutics, ultimately supporting the development of safe, effective, and economically viable treatments for various diseases.

## 1. Introduction

Virus-based therapeutics is a rapidly evolving field, leveraging the unique properties of viruses to treat a variety of diseases. These therapeutics include vaccines against infectious diseases and cancer, gene therapies, and oncolytic therapies, each with its unique mechanism of action and therapeutic potential [1].

Vaccines, the most well-known type of virus-based therapeutics, use viruses or parts of the virus particle to stimulate the body’s immune response. This concept of immunization to prevent disease dates back to the late 18th century when Edward Jenner developed the smallpox vaccine. Nowadays, conventional vaccines use live-attenuated or inactivated pathogenic viral particles or viral subunits. More recently developed vaccines, primarily due to the pandemic outbreak of coronavirus, utilize viral vectors or vehicles to deliver the genetic information to cells for expression of viral subunits. Vaccination strategies that prevent oncovirus infections and, thereby, prevent infection-related cancers are considered cancer vaccinations as they mitigate the cause of tumor formation [2].

Gene therapies use virus particles (VPs) as vectors to deliver therapeutic genes into patients’ cells, offering potential treatments for genetic disorders. The first approved gene therapy was alipogene tiparvovec, marketed as Glybera, approved in Europe in 2012 for the treatment of lipoprotein lipase deficiency [3]. Due to increased malignancy risks correlated to the replication-competency of viruses, VPs for gene therapies are engineered to transduce genes but not to replicate or regain replication-competency [4].

Oncolytic virus (OV) therapies, on the other hand, employ replication-competent viruses that preferentially affect cancer cells over normal cells, leading to the lysis of tumor cells and the stabilization and reduction of tumor progression [5]. Surface-presented proteins on VPs confer their tropism for specific cells in the host, and they can be redirected to other cells of interest by manipulation through genetic engineering of the virus. In addition to direct tumor cell lysis, OVs can stimulate the immune system to mount an anti-tumor response. This is achieved by modifying the tumor microenvironment from an immune-tolerant state to an inflamed state, enabling the immune system to effectively target and kill abnormal cells [6]. This mode of action is known as cancer vaccine treatment and can be enhanced by inserting transgenes into OVs to modify viral competencies or to specifically modulate the host response [7].

OVs should be capable of replicating within the host’s cells while being attenuated to prevent pathological infection. The advantage of using replication-competent viruses is that they can amplify their numbers within the tumor, leading to the spread of virus infection and increased expression of transgenes. Replication-competency is a key factor for OVs to facilitate an enhanced therapeutic effect. The first FDA-approved OV therapy was Talimogene laherparepvec (T-VEC) in 2015 for the treatment of melanoma. T-VEC is an example of a replication-competent OV, which is a modified herpes simplex virus (HSV). In this example, neurovirulence genes were deleted to improve safety and efficacy, while added immunomodulating genes enhanced the host immune response [8].

Of the 103 active clinical studies involving OVs at the beginning of 2025, 65% were utilizing enveloped VPs (env. VPs) [9]. The outermost layer of env. VPs is a lipid bilayer derived from the host cell. This envelope embeds viral proteins, predominantly glycoproteins, equipping the VPs with special properties useful for therapeutic applications. Envelope proteins responsible for cell recognition and attachment can be replaced, modified, or added, generating pseudotyped viruses. This pseudotyping of replication-competent env. OVs are typically performed by genetic engineering. For non-enveloped VPs, the highly structured capsid layer constrains the incorporation or modification of surface structures. Capsid engineering requires more sophisticated techniques for tropism modifications without compromising the capsid integrity [10]. Another reason for the widespread use of env. VP for therapeutic applications is the large capacity for gene inserts [11]. However, envelope VPs have inherent drawbacks mostly related to their labile lipid envelope structure and its variability, presenting challenges for bioprocessing, which will be elaborated in Section 1.1.

Manufacturing requirements for advanced therapy medicinal products (ATMPs), which include virus-based therapies, are formulated by regulatory agencies in several guidelines [12,13]. Drug product quality attributes such as identity, purity, potency, and safety might be impacted by the bioprocess and need to be assessed to ensure a safe and effective application of viral therapeutics [14]. Therapeutic products based on replication-competent env. VPs constitute an especially labile drug substance. Live attenuated vaccines and oncolytic VPs both fall into this category, but oncolytic VPs are subject to stricter specifications. This combination of sensitive drug substances and high requirements poses challenges to biomanufacturing and the purification process in particular.

This review focuses on the purification of replication-competent env. VPs with therapeutic applications. A selection of viruses within this scope that are currently in development is shown in Table 1. The status quo regarding current challenges, best practices, and the impact of process steps on the integrity of viral particles are evaluated using published references. However, reviews, case studies, and protocols are not always available for this set frame. The bioprocessing literature landscape for replication-deficient VPs and virus-like particles (VLPs), enveloped or non-env. VPs lacking therapeutic applications is substantially broader. For these products, analytical methods and purification processes are often simpler and more widely implemented due to their less complex particle structures and lower processing demands. However, where transferability between virus modalities could reasonably be inferred, references from other viral modalities were included and discussed.

### 1.1. Challenges in the Purification of Enveloped VPs

The complexity and non-rigid structure of the lipid membrane makes env. VPs vulnerable to perturbations of optimal environmental conditions, such as elevated temperatures [25,26,27,28], pH [26,27,28,29], osmolarity, and ionic strength [30]. Solvents, detergents [31,32], and excipients, such as arginine, might impede viral activity [33]. Previously exploited for viral inactivation [31], these conditions must be avoided in purification approaches. Furthermore, freeze/thaw-cycles (FT-cycles) [34,35] and shear forces [27,28,36] can cause VP degradation, leading to a decrease in infectious titers while increasing impurity content. Furthermore, sample handling and conditions during analytical methods can skew analytical results [28,37]. Forced degradation studies may help to find conditions that maintain viral integrity [27,38]. In general, the processing time and number of steps should be minimized. Purification processes might also be conducted in a cooled environment, but scalability issues are apparent [39].

The lipid membrane is variable in the composition of lipids and viral and host cell proteins (hcPs) [40]. Differences between virus species, strains, serotypes, and engineered virus variants are evident. Structural differences between virus types exist in size, morphology, and number of envelopes. The heterogeneity and complexity impede the development of efficient platform processes. Hence, process development efforts often start anew if process knowledge is not available or methods cannot be transferred. Genomic variants, established via evolution or genetic modifications might also require adaptations of process parameters [41]. Heterogeneity of VPs also prevails between production batches due to direct dependency on the cell culture, which is subject to variability and, furthermore, leads to heterogeneity of particles within the same batch [42,43,44,45].

In biopharmaceutical processes, impurities from cell culture, such as whole cells, hcPs, and host cell DNA (hcDNA), need to be depleted. However, during the budding of env. VPs, viral proteins as well as host cell-derived proteins are incorporated either in the tegument or the envelope [46,47,48]. Some incorporated cellular proteins may contribute to viral replication, which complicates the definition of the target product profile [46,49,50].

Impurities of the same size range as the VPs, such as extracellular vesicles (EVs), are more complex to separate [51]. EVs are membrane particles secreted naturally by cells in various sizes and with diverse enclosed content and membrane proteins. Viruses and EVs use overlapping cellular pathways which results in a high similarity between them regarding structure and protein/DNA content [52]. EVs’ biological function and their role in viral infectious is a topic of ongoing research. Some EVs are shown to facilitate viral infection, while other EVs carry anti-viral features [53]. Increased EV production upon cell infection was observed for multiple virus strains [54]. This class of impurities is difficult to assess and it is unclear if it should be regarded as impurities [51].

By comparing the count of non-infectious VPs (non-inf. VP) to infectious (inf. VP), the particles-to-infectious unit ratio (P:IU) can be derived. Corresponding analytical methods are discussed in Section 2.1. A longstanding challenge in virus bioprocessing is the population of non-inf. VPs that usually predominate the material generated [55,56]. Non-inf. VPs lost, or never gained, their functional integrity due to malformations or errors in composition. The initial amount of non-inf. VPs is dependent on host cell type and upstream conditions [45,57,58]. In non-clinical efficacy and safety-studies, usually only infectious titers are reported. Correlations of side effects with non-inf. VPs can, therefore, not be made. At the same time, viral therapies have been applied and clinically tested for decades and are generally considered safe [59]. The practical approach to purifying VPs aims to minimize the presence of non-inf. VPs. From a processing perspective, EVs and all non-inf. VPs reduce the efficiency of purifying the desired infectious VPs [60].

Dosages of live attenuated vaccines are in the range of 10^4^ to 10^8^ infectious units (IUs) per dose [61], whereas the dosages for enveloped OVs reach up to 10^11^ IU per dose for intravenous applications [62,63]. High dosage requirements for OVs at the drug product level necessitate the processing of high volumes and efficient concentration due to usually lower titers at harvest level. During the whole bioprocess, the functional integrity which is key for the replication-competency and, thus, therapeutic effect, must remain intact. Ideally, non-infectious particles are reduced together with the depletion of immunogenic impurities such as hcP and hcDNA.

The size of env. VPs enables the insertion of transgenes for therapeutic applications but also increases their complexity and sensitivity to environmental stress and poses a challenge for the bioprocess. Conventional methods for processing biopharmaceuticals are primarily designed for protein molecules, like antibodies, which are significantly smaller in size. Other novel chromatographic and filtration approaches are used to overcome these size limitations. Experience and knowledge applying these methods are currently generated. Unexpected effects arise, such as convective particle entrapment (see Section 7.2.3), multi-point binding of VPs (see Section 7.1.5) and irreversible time-dependent binding (see Section 7.1.5), for which mechanistic insights and solutions are needed.

In addition to the aforementioned challenges directly involved with the bioprocessing of env. VPs or VPs in general, analytical methods to evaluate VP preparation are limited. As further discussed in Section 2, the available analytical panel lacks accuracy, high throughput, and quick turnover time.

### 1.2. General Purification Scheme for Therapeutic Enveloped VPs

Replication-competent VPs are propagated by infection of permissive production cell lines. Sophisticated bioreactors, simple shake flasks, or supports for adherent cell lines are used for cell culture in which the cells are provided with the necessary nutrients and conditions to grow and produce. Once the cells have reached a target density, they are infected with a replication-competent virus at a specified multiplicity of infection (MOI) ratio. The virus uses the cells’ machinery to replicate itself, producing virus progeny.

**Figure 1:**
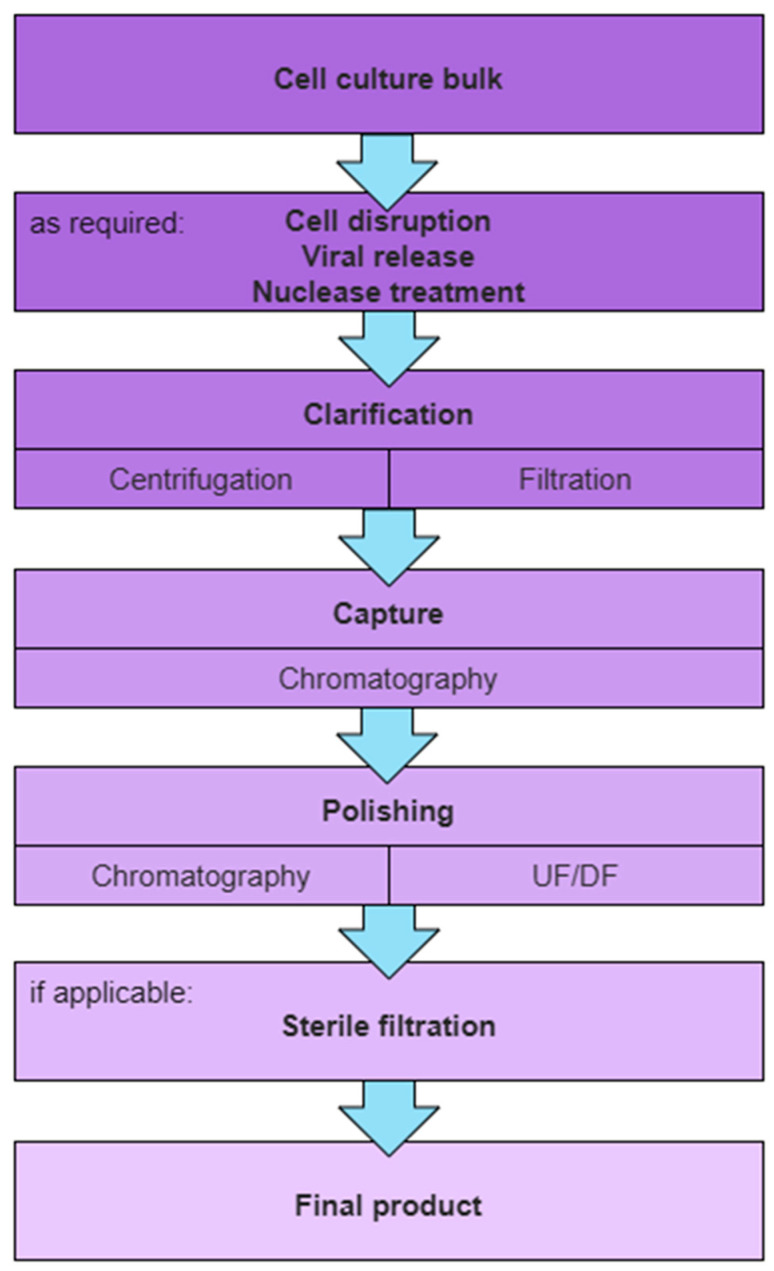
Generalized purification scheme for replication-competent enveloped VPs.

A general purification scheme for VPs intended for clinical grade material production, as shown in Figure 1, is the following: clarification, capture, polishing, and sterile filtration. Further unit operations may be included as required, dependent on the virus life cycle, harvest strategy for the VPs, and target purification requirements.

Prior to a clarification step, cell disruption, viral release, or a nuclease treatment may be performed to improve initial VP titers and VP recovery. Cell disruption aims to break up cells to release intracellular VPs to render them accessible for purification. VPs might be non-covalently attached to cells or cell debris and thus cell-associated. A viral release step involving the addition of additives (e.g., salt) can facilitate the release of VPs into the supernatant. The addition of nucleases degrades free DNA and RNA chains and also reduces viscosity, which improves the processability of the harvested material.

Initial clarification of harvest material is essential to remove solids in the feed stream, such as cells, cell debris, and aggregates, all while ensuring the maximal recovery of intact VPs. Techniques such as centrifugation and filtration are commonly employed. At lab scale, the clarification is often divided into primary and secondary clarification, providing a robust setup for the removal of, first, large particulate matter, such as intact and non-viable cells, and, subsequently, colloidal matter, such as large aggregates, without exceeding device limitations.

The capture step aims to selectively concentrate VPs from clarified harvest, effectively separating them from the bulk of process-related impurities and reducing the process feed volume. Chromatographic modalities such as ion exchange chromatography (IEX), hydrophobic interaction chromatography (HIC), and affinity ligands confer specific binding to VPs while most impurities are in the flowthrough. To maintain reasonable processing times with high volumetric feeds, high flow rates are necessary, making convective chromatographic media like membrane and monolithic stationary supports preferable.

The polishing step is designed to further reduce remaining impurities to achieve the low levels demanded by regulatory bodies for clinical application. A buffer exchange into the formulation buffer is typically applied at this stage as well; thus, flowthrough (FT) chromatography, size exclusion chromatography (SEC), or ultrafiltration and diafiltration (UF/DF) are applied. Subsequently, stabilizers or other additives might be added to ensure the VPs remain stable and effective.

The final step is to sterilize the VP preparation to remove any remaining contaminants. In case conventional microfiltration is not applicable due to the size of VPs, the entire bioprocess must be conducted under aseptic conditions.

## 2. VP Integrity and Analytical Methods for Its Assessment

VPs comprise a genome (DNA or RNA) bound by capsid proteins and additional viral-encoded proteins. Env. VPs are additionally surrounded by a lipid bilayer derived from the host cell membrane that incorporates virus- or host-cell-derived and potentially glycosylated proteins [64]. This envelope is not merely a protective layer; it is integral to the virus’s morphology and stability.

The envelope and the correct assembly of all viral components, such as the capsid and the viral genome, provide the framework for a functioning replication-competent VP. The physical intactness of all VP components and structure is termed structural integrity. Any damage or alteration to the structural integrity can affect the virus’ ability to infect host cells and replicate and, thus, influence the responses they provoke in both in vitro and, notably, in vivo experiments. The components also account for the stability of the structural integrity and are, therefore, virus-specific, whereas env. VPs are particularly labile due to their envelope, as discussed in the introduction.

Functional integrity, on the other hand, refers to the virus’ ability to perform its intended functions. This includes the ability to bind to host cells, enter these cells, replicate its genome, express viral proteins, assemble new VPs, and exit the host cell. As part of the infectious cycle, proteins encoded on the genome are expressed, including introduced transgenes with therapeutic effects. Any disruption of listed functions can significantly impact the virus’ infectivity and, thus, its efficacy as a therapeutic agent.

For bioprocessing, maintaining the structural and functional integrity of env. VPs is critical to ensure high infectious titers. Understanding and monitoring viral integrity is an important aspect during the development of purification strategies for VPs. This knowledge can help improve the production process, ensure the quality of the viral products, and, ultimately, contribute to the development of safe and effective viral therapies and vaccines.

### 2.1. Quantification Methods

For virus process development, in-process control samples are taken to assess virus yield, depletion of host cell impurities, and presence of contaminants (not part of this review). Yield of env. VP processes are typically assessed by measuring both inf. VP titer and total VPs (inf. VPs + non-inf. VPs), which together are informative regarding the influence of the unit operation on the recovery performance and its influence on VPs functional integrity. The derived P:IU ratio depends on the specific analytical methods used to determine the individual values. For total VP count, several methods exist; each method targets a specific property or particle characteristic, e.g., genomic content, light scattering events, or antigen content. Hence, results can differ, and population overlaps between different analytical methods are difficult to derive. The specific methods and their limitations should be considered in the evaluation of the P:IU ratio. Most quantification methods can also be applied for non-enveloped VPs, and additional methods exist to determine the full/empty state of particles. Lothert et al. recently reviewed and evaluated available quantification methods for VPs to which the reader is referred for more information regarding the individual analytical methods [65].

#### 2.1.1. Infectious Titer Assays

Infectious titer is conventionally measured by end-point cell-based assays: 50% tissue culture infective dose assay (TCID_50_) or plaque assay-determined plaque forming units (pfu). These assays are straightforward to perform and do not require sophisticated equipment. Although they are applicable to different viruses, the actual setup must be adapted for each virus to be measured (especially permissive cell line and incubation time). However, they rely on multiple rounds of viral replication, which may take up to a week for evaluation and are sensitive to operator handling [65]. Generally, a TCID_50_ variability of 0.5 log steps is reported [66,67]. Contemporary applications of the TCID_50_ method reduce hands-on time by using pipetting robots and digital image analysis for the evaluation of infected cells. The approach reduces variability to approx. 20% [68].

Other faster and more sensitive infectivity measurement methods have been developed. These require only one round of replication and quantifying the inf. VP titer against a calibration curve. Cytopathic effects in the cell culture may be quantified based on changes in cell morphology by imaging, and reach variabilities of below 20% [69]. Alternatively, fluorescent markers, either expressed in infected cells or by antibody stain labeling, can be measured with flow cytometry or imaging [70]. However, these enhanced infectivity methods are more technically challenging and require specialized equipment.

#### 2.1.2. Total Virus Particles

Total VPs can be measured by quantifying viral antigens and viral genome, and by particle analysis. Structural viral antigens may be measured using immune techniques such as enzyme-linked immunosorbent assay (ELISA) or indirectly using hemagglutination (HA) and radial immunodiffusion (RID) assays [65]. These assays provide same-day results, but they require specialized substrates and a standard curve for quantification. For enveloped viruses, these methods may overestimate actual virus titers due to potential variation in the amount of incorporated viral proteins on the outer lipid membrane dependent on upstream conditions as well as the method of clarification. Viral genomic copies can be measured by polymerase chain reaction (PCR). Upon extraction of the viral DNA or RNA, the method of choice is quantitative PCR (qPCR) using a suitable pair of primers and detecting the amount of amplification by measuring fluorescent intensity after each cycle. Genomic copies are quantified based on a calibration curve using a known standard. Further advanced PCR methods, such as digital PCR and digital droplet PCR (dPCR and ddPCR), can directly quantify the viral genome without reliance on a calibration curve and may be performed with less sample handling. Digital PCR methods are characterized by improved precision, with a report showing a 5-fold improvement in precision and a 20-fold improvement in accuracy for human immunodeficiency viruses (HIV) genomic copies analyzed by ddPCR compared to conventional qPCR [65,71]. PCR methods can reliably quantify viral genome copies, but for env. VPs, they potentially overestimate total VPs as not all viral genomes detected are incorporated into VPs with full functional integrity. Furthermore, dependent on the amplicon chosen for the assay, it does not distinguish between complete and truncated genomes. A multiplex ddPCR result for (non-enveloped) adeno-associated viral vectors (AAVs) showed a discrepancy of 40% between amplicons used due to AAVs containing incomplete genomic copies [72].

#### 2.1.3. Total Particles by Light Scattering (LS)

Total particles can be rapidly quantified by LS techniques using both dynamic and static LS (SLS). Such techniques require a laser beam directed at the sample, and fluctuation of scattered light based on the Brownian motion of particles, as well as the intensity of the scattered light, are detected [65]. Dynamic LS (DLS) and nanoparticle tracking analysis (NTA) are both methods that rely on the detection of the Brownian motion of particles in solution to ascertain the size distribution of a particle population. While NTA tracks individual particles using microscopy lenses, the DLS indirectly calculates the particle count based on the intensity of the scattered light. Both methods are vulnerable to drift motions in the measurement chamber and the influence of solvent composition, and their particle concentration range for measurement is limited [73,74]. Furthermore, for polydisperse samples, especially those containing impurities of large sizes and aggregates, the result can be skewed. The increased scattering intensity of larger particles can lead to an underestimation of smaller particle populations, and results depend highly on chosen device settings [75,76]. Thus, measurement parameters must be tightly controlled for the precision of the result. Multi-angle LS (MALS) is an advanced method using SLS at different angles to obtain information on particle size, shape, and molecular weight, as well as particle count. It is primarily used in conjunction with a separation technique such as chromatography or field-flow fractionation (FFF) [77]. However, the full potential of a MALS analysis is often not reached for enveloped viruses due to the heterogeneity of VPs and sample composition, as well as unknown particle properties such as the refractive index (RI) increment.

LS measurements are non-invasive and can provide rapid results, making them suitable for real-time measurements during a process [77] and as a quality control method. A reported quantification method using FFF-MALS showed good accuracy and precision (<5% and <2%) [78]. However, LS methods indiscriminately consider all particle types present in the solution, env. VPs and also EVs, and the extent of such overestimation of total env. VPs depends on upstream conditions as well as the purification stage of the sample. The separation capability of current analytical assays is insufficient to separate EVs from VPs [51].

### 2.2. Structure and Composition

The structural integrity of VPs is defined by their intact structure and correct composition of components. The composition of env. VPs is determined not only by the viral genome but also by the production cell line and the specific upstream conditions [61]. The virus structure can be evaluated by a combination of advanced imaging techniques, assessment of viral envelope, as well as X-ray crystallography (used for viral research). Mass spectrometry (MS), capillary gel electrophoresis (cGE), and Western blotting are common methods to look at particle composition. Recent developments adapting flow cytometers for the detection of viruses gave rise to the method referred to as flow virometry. It is a rapid method that allows for the detection and analysis of single VPs in a bulk sample based on its known characteristics like size, surface structure, and composition [79].

#### 2.2.1. Structure

To understand the effect of a unit operation on VPs, high-resolution images that can facilitate a close examination of VP structure and substructure on a single particle level are essential. Of particular interest is to look at samples taken before and after a process step for the proportion of intact particles versus degraded particles, empty vesicles and EVs, and VP aggregates. Electron microscopy (EM) can facilitate the capture of such images, either by cryogenic EM (cryo-EM) looking at snap-frozen VPs in a hydrated state or by negative staining EM (nsEM) in a dehydrated state [64]. Cryo-EM can be performed on VPs in their native state in various conditions, requires a small amount of virus, and is suitable for viruses that take up a symmetric shape and also irregular shape, albeit at a lower resolution [80]. Studies of dengue virus (DENV) by cryo-EM revealed the influence of temperature on the conformation of the envelope [81]. NsEM can reveal in detail VP substructures and also surface protrusions from enveloped incorporated glycoproteins [82].

Structural variability of env. VPs is first introduced upstream during virus budding, reflecting the host cell line, the state of the cells after infection, and the specific virus budding mechanism [80]. During a unit operation, VPs can be (partially) degraded (degradation of surface glycoproteins, leakage of nucleocapsid, irregular shapes) and form small aggregates. While some degraded particles and VP aggregates retain infectivity, as seen in infectious titer measurements, they elicit unwanted effects during in vivo experiments [61]. Detection of undesired particle degradation through high-resolution imaging is crucial for the evaluation of the quality of the target VPs during production.

While EM imaging can provide detailed structural information on single VPs, as well as detect the presence of non-VPs, the technique is accessible only in specialized labs and can be performed only on a limited number of samples. LS techniques analyze whole VP samples to obtain size distribution information, which can be useful for detecting significant particle degradation as well as aggregation. Correlations between the resulting size distribution and EM imaging results may provide quick insight into the potential detrimental effects of a unit operation on VPs [83].

#### 2.2.2. Composition

For therapeutic applications, viruses are often engineered with exogenous sequences inserted into the genome, while viral genes that confer undesired pathogenicity are deleted [84,85]. The genomic stability of the target virus should be examined, especially during upstream parameter optimization (e.g., multiplicity of infection (MOI), timepoint of infection (TOI), and timepoints of harvest (TOH)). While PCR-based methods and Sanger sequencing are readily accessible techniques to examine specific genomic stretches, next-generation sequencing (NGS) and deep sequencing methods provide a more comprehensive view of the entire genome.

The composition of viral and acquired cellular proteins reflects cell culture conditions and is indicative of its function in terms of infectivity and potential subsequent immunogenicity. Characterization and absolute quantification of protein content, including post-translational modifications, was performed for a vesicular stomatitis virus (VSV) pseudovirus by multiple reaction monitoring MS [86]. A number of host-derived proteins have been characterized for their function in the virus replication cycle, such as chaperones for protein folding, complement control proteins, vesicular transport proteins, and adhesion molecules [46]. Relative quantification techniques by MS would be instrumental in not only the identification of such incorporated host cellular proteins but also their differential proportions relative to viral proteins for samples taken during upstream optimization [87,88]. Further, the lipid and glycoprotein content of env. VPs should be examined, especially when choosing a production cell line. The envelope’s fluidity and, thus, its stability and integrity were shown to depend on the host cell line [89]. Envelope-incorporated glycoproteins impact the stability, shown in increased resistance against shear forces in process unit operations [90,91]. Kim et al. observed a higher functional stability for retroviruses pseudotyped using the VSV-G glycoprotein compared to pseudotyping with an influenza envelope protein [91]. Specific to the virus life cycle, the lipid membrane may be derived from intracellular structures, the plasma membrane, and sometimes it is derived from a distinct membrane region such as lipid rafts. Additionally, the glycosylation pattern of lipids and incorporated glycoproteins may differ between different cell lines, as identified using matrix-assisted laser desorption/ionization MS (MALDI-MS) upon lipid isolation [92]. These attributes have direct consequences on VP stability in solution and its reception in vivo. As MS techniques are not ubiquitously accessible, Western blotting and cGE can be used for virus identification and estimation of the number of individual proteins for in-process control samples taken during process development [93,94].

#### 2.2.3. Flow Virometry

Flow virometry is a recently developed technique that facilitates the examination of viral particles (VPs) at a singular particle level, providing insights into their size, structural integrity, and biochemical composition. This method hinges on the specific labeling of VP components in a solution, followed by the passage of these particles through a detector [95]. Simultaneous labeling of VPs facilitates a multiplexing approach [96]. The technique is potentially of high throughput while generating rapid results, and it can be set up in process development labs as an at-line assay. Not only can it provide insight into particle concentration and purity (quantification of non-viruses), but it can also detect VPs that are degraded during a production process [97,98]. With such real-time insights, this technique facilitates the improvement of production quality and yield overall. Moreover, flow virometry can also be used to evaluate host antibody-mediated immunogenicity against the VP preparation [99].

#### 2.2.4. Mass Balance for Virus Process Development

For virus process development, mass balance is essential though challenging to achieve. This is due to the complexity of measuring specific components in a complex biological system, as well as the dependency on biological assays to measure functional integrity. Measuring all components in an in-process control sample accurately and simultaneously requires advanced analytical techniques. Recent developments in analytical separation techniques such as SEC and FFF coupled with multiple online detectors (including UV, RI, DLS, and MALS) facilitate mass balance measurements. VPs can be quantified in a bulk sample with good accuracy and precision. These methods are especially useful for enveloped virus (or virus-like) particles that are not replication-competent for accurate particle quantification [65]. Further, this approach can be used to quantify protein and nucleic acid components in complex samples due to the separation from smaller process impurities. It may be used as a quality control method, and also as an at-line measurement during a production process or process development.

For non-enveloped coxsackievirus, a method for VP characterization including quantification of drug substance and process intermediates has been developed based on SEC separation [100]. In another study, an FFF-MALS characterization method was established for the influenza virus as a vaccine candidate [78]. The reported precision for both methods was <2% and, therefore, is very useful for mass balance applications, though such use cases were not applied. In a similar quantification method for a VSV pseudovirus, the mass balance of a chromatographic unit operation was performed, and the virus content in the samples was quantified using an HPLC-SEC setup. The reported method precision was <3%, which resulted in an overall mass balance discrepancy of about 15% [37].

### 2.3. Further Commentary

Process development, being upstream optimizations or downstream endeavors, can only be pursued if the outcome of process changes can be evaluated. Analytical methods are required to characterize process steps in regard to the structural and functional integrity of VPs as well as the preparations’ purity. Ideally, analytical methods are high-throughput, accurate, precise, and deliver same-day results. Currently used and well-established methods do not fulfill these requirements. Many different methods exist, each with its own drawbacks, e.g., low VP specificity for NTA and DLS measurements, tedious and low precision for infectivity assays such as TCID_50_ and plaque assays, or skewed PCR results due to the counting of free or EV-associated DNA or RNA [65]. To gain a complete picture despite the mentioned drawbacks of current analytical methods, an analytical panel of various methods is typically used at the moment. However, performing such a panel is time-intensive and is not conducive to making fast decisions during process development.

Furthermore, analytical methods cannot be easily transferred between different viral particles. Methods need to be developed, validated, and established before usage. Here, analytical development and process development are co-dependent and represent a ‘chicken-or-the-egg’ dilemma, with process development requiring established analytical methods and analytical development requiring purified and well-characterized material. Thus, both developments should be conducted in parallel.

Ideally, the results of multiple assays can be allocated to particle populations and, thus, subpopulations distinguished by various properties quantified. This would enable a comprehensive evaluation of particle integrity and its correlation with functional integrity. Distinct properties such as glycoprotein content, genomic content, morphology, and infectivity would need to be allocated. This requires either multidimensional assays or assays capable of multiplexing on a singular particle level. Multiplex approaches utilizing microfluidic devices and immunostainings (ELISA or flow virometry [96,101]) and multiplex qPCR assays [72] exist. However, simultaneous detectable properties are limited and insufficient for the afore-drawn ideal case. Furthermore, these analytical methods are complex and not ubiquitous. Hence, only individual properties within the whole population can be evaluated and their change within the process monitored.

## 3. Cell Culture and Infection

Replication-competent VPs are produced by propagation in a susceptible production host cell under optimized culture conditions [102]. The lipid bilayer of env. VPs is derived from the host cell, and thus, its structure and composition depend on the cell line. While this does not necessarily impact infectivity [89], virion structural integrity relies on the envelope composition.

Current viral vector production processes rely on both adherent and suspension cell cultures in chemically defined media. While technologies exist to upscale both culture platforms, suspension culture is advantageous for the operator due to easier handling and scalability. However, bioreactors induce shear forces through agitation and aeration, for example, stirred tank reactors (STRs) that utilize impellers and gas spargers [103]. VPs that are released extracellularly in the culture are exposed to the resulting shear stress and may be negatively impacted. Grein et al. observed a high impact of aeration and agitation on measles virus productivity in a STR for Vero cells using microcarriers [104]. The increase in aeration in response to elevated oxygen consumption of infected cells resulted in a 4-log reduction in viral titers. The authors suggested the utilization of bubble-free aeration methods, which were shown to work for high oxygen-demanding cell cultures [104]. Another type of microcarrier was introduced by Yekrang Safakar et al. which locally shields cells from shear stress by a hollow structure. While the structure’s benefits were shown for sensitive stem cell cultivation [105], the impact on viral productivity has yet to be shown. New impeller concepts utilizing a flexible, multiple impeller setup aim to reduce introduced shear stress while keeping sufficient agitation and simple scalability [106].

Infecting the cell culture at the late exponential (G2M) phase of the cell cycle typically yields the highest infectious titers for enveloped viruses [107,108]. The ratio of applied inf. VPs-to-viable cells is described by the MOI. Upstream parameters, including TOI, MOI, cell density, and time of harvest (TOH), are interlinked, and remain to be optimized to produce each virus according to its specific virus life cycle [109]. While a low MOI reduces the required amount of the master seed virus, a high MOI usually results in an earlier harvesting time point [110]. Especially for enveloped RNA viruses, VPs with truncated genomes of variable sizes, namely defective interfering particles (DIPs), naturally occur and can spontaneously arise in culture [111]. The DIPs are by-products of virus production that share structural similarities with replication-competent VPs but contain a significantly truncated genome. They cannot replicate on their own but compete with standard VPs for replication in co-infected cells, thereby reducing the infectious titer, and should be monitored in virus production. By using low MOIs, the likelihood of co-infections is reduced, and reports show a lower DIP count [112].

Developments toward process intensification demonstrate a positive correlation between virus titer and cell density at TOI [113,114,115], using both simple fed-batch or sophisticated perfusion systems using cell retention devices. Gutiérrez–Granados et al. provide a good overview of perfusion culture systems in their review of process intensification methods for viral vaccine and viral vector production [116]. However, the impact on viral integrity was not evaluated.

## 4. Harvest of Viral Particles

At time of harvest (TOH), VPs are collected from the host cell culture and fed into the subsequent purification process. Harvesting conditions, such as TOH and cell disruption methods, impact viral integrity and impurity content and, thus, have an influence on the overall process performance.

Virus-infected cells produce viral progeny until cells eventually die due to the overuse of metabolic pathways for viral replication or induced viral cytopathic effects, e.g., apoptosis by VSV infection [117]. For bioprocessing, harvesting of extracellular VPs should be performed prior to the exponential cell viability drop to reduce the impurity burden on purification steps while maximizing yield. However, from a biological point of view, the optimal TOH needs to be evaluated in terms of viral integrity. The condition of an infected cell’s culture at early and late stages influences viral integrity and composition, as seen by their release of different amounts of infectious and non-inf. VPs as well as EVs [45,118]. Furthermore, morphological and functional differences are observed for early (24 hpi (hours post-infection)) and late (≥48 hpi) harvested paramyxovirus particles cultivated in eggs [119]. The mechanistic understanding of the cellular state after infection and the viral integrity of VPs remains a knowledge gap. Hence, VP quality and concentration, as well as impurity levels due to dying host cells, need to be considered and evaluated to find the cell culture and virus-specific optimal TOH. Dielectric spectroscopy has been applied as an online detector for frequency-dependent capacitance measurements, and thus, cell culture state and level of infection could be derived in real time [120]. It proved to be useful to support TOH optimization efforts and real-time control of the optimal TOH.

### 4.1. Continuous Harvest

Infectivity loss of VPs over time in a production culture has been shown in a bioreactor. The elevated cell culture temperatures and exposition of VPs to proteases and shear stress in the cell culture are presumed to reduce the infectivity of influenza and measles VPs [121,122,123]. Perfusion systems, initially developed for process intensification, have been explored for continuous extraction of VPs. The continuous harvesting approach improved cell-specific productivity for influenza VPs by a factor of four compared to a batch process [121]. At the same time, inhibitors of the viral replication are removed, and depleted nutrients are replaced, which was shown to keep viral productions high (e.g., for VSV in HEK293F cells [124] and HSV-1 in Vero cells [125]).

Membrane-based cell retention systems have been successfully utilized in different modalities for the continuous harvest of env. VPs, including tangential flow filtration (TFF) [126], alternating TFF (ATF) [127], and tangential flow depth filtration (TFDF) [115]. A recurring issue of the membrane technology is membrane fouling and, thus, loss of perfusion functionality. High optimization efforts are required to establish a suitable system for cell retention and continuous viral harvest without early membrane fouling. Non-membrane devices such as acoustic settlers do not suffer from fouling issues. An influenza perfusion cell culture utilizing an acoustic settler showed a 1.5 to 3 times increased volumetric productivity compared to an ATF perfusion device [122]. The comparison of the acoustic settler perfusion process to a batch process for a purification-challenging fusogenic VSV pseudovirus showed an increase of factor 15 to 30 in volumetric productivity [128].

The prerequisite for a continuous harvesting approach is a prolonged harvesting window before the viral cytotoxic effects dominate, leading to a high impurity content at low additional productivity. Non-destructive virus budding exists also for some enveloped viruses, allowing an extended virus production phase in the host cell. Both possibilities enable a continuous viral production (e.g., vaccinia on AGE1.CR.pIX [129]) or several rounds of harvesting [45], which can be exploited in perfusion reactors (HSV-1 on Vero cells [125] and VSV on BHK-21 [115]).

Continuous or repeated harvest during a perfusion culture may not necessarily improve yields significantly to justify the increased development and processing effort, as shown for a VSV perfusion culture [124] and temperature-stable modified vaccinia Ankara (MVA) VPs [130]. Additionally, as discussed in the introduction to this section, the VP quality depends on the TOH and needs to be evaluated (P:IU changes for mumps and measles VPs [45]).

Online monitoring of processing parameters, e.g., by real-time process analytical technologies (PAT), is especially useful for continuous processes to control the process and derive real-time process and quality attributes. In VP processes, it is difficult to derive real-time information from online detectors due to the complex VP structures, VP heterogeneity, and similarities to impurities [131]. Referenced studies in this section, thus, relied mostly on the application of at-line or offline analytical methods to evaluate cell culture conditions and product quality. However, it was shown that the application of an online dielectric spectroscopy detector could provide not only the state of the cell culture in real-time but also valuable information about the state of infection in cell cultures infected by env. VPs [101,120]. Different dielectric frequencies were used to distinguish between capacitance measurements for cells and VPs, enabling the estimation of VP count. An application to continuous harvesting approaches could increase process robustness through process control and provide real-time estimation of VP count. Gränicher et al. used capacitance measurements to monitor cell culture parameters, control perfusion conditions, and optimize TOH for vaccinia release [129]. However, the precision of the release timepoint was low (±4 h), and no VP count estimation was derived from capacitance measurements. More studies evaluating the use of online dielectric spectroscopy are required to determine the usefulness for env. VP processes.

### 4.2. Cell Disruption

During the viral replication cycle, env. VPs acquire a host-derived lipid membrane through a budding process from host cell membranes. Intracellular and extracellular budding exist, whereas the latter budding results in a cellular egress [132]. Viruses being developed for therapeutic application that first undergo intracellular budding include poxviruses [133] and HSV [134]. Intracellular budding viruses go through several intermediates within the host cell, distinguished by shape and the number of acquired lipid membranes. Mature particles can egress by exocytosis or after cell lysis, while the intermediate stages can already be infectious and accumulate in large numbers, as shown, e.g., for vaccinia [135]. Thus, at the time point of harvest, cell-disruption methods can be applied to increase VP titers. It is worth noting that the viral intermediates differ in surface protein composition, which impacts viral attachment and entry, as observed for multiple poxviruses [136,137]. To our knowledge, there is no published study evaluating the functional integrity of derived subpopulations and their effects on therapeutic applications.

Cell-disruption methods include manifold mechanical methods, as well as chemical lysis used to release viral particles from cells. At the lab scale, a combination of FT-cycles and sonication is commonly used to increase harvest recoveries for VPs (e.g., HSV-1 [138], Orf [139], and Newcastle disease virus (NDV) [140]). The formation of ice crystals impairs the stability of the cellular lipid bilayer. At the same time, FT-cycles also impact the viral integrity of env. VPs (herpesvirus [141], retrovirus [35], VSV [34]). Furthermore, FT-cycles are impractical for large-scale production volumes.

Sonication as an alternate method of mechanical lysis is scalable with flow-through probe devices [142]. However, sonication induces shear forces due to cavitation, which results in cell disruption, a phenomenon that may also negatively affect env. VPs. This is observed in surrogate VPs over extended time frames [143]. The power densities and exposure time required for cell disruption are notably lower than those applied to the surrogate VPs. In a stability study for orf particles, a decrease in infectious titers was observed within minutes of sonication, though the loss may be attributed to the uncooled environment [38]. It is imperative to maintain temperature control of the material and to minimize the induced energy to mitigate potential detrimental effects on viral integrity.

Homogenizers are another cell disruption category with a multitude of devices and scale-up possibilities available [142]. Mundle et al. compared sonication and microfluidization methods for Vero cell disruption and harvest of respiratory syncytial virus (RSV) particles [144]. A similar increase in infectious titer could be achieved for sonication and low-pressure microfluidization of harvest, whereas repeated or higher pressure microfluidization showed a lower or decreased infectious titer. With both methods, an expected increase in soluble host cell impurities was observed [144]. The question of the structural impact of the cell disruption methods was not evaluated, although mechanical stress has been shown to generally impair the envelope of viral particles [119].

Cell disruption by osmotic pressure (hypotonic conditions) is a mild method to release intracellular components. Laposova et al. showcased the beneficial use of deionized water on BHK-21 cells for disruption and release of lymphocytic choriomeningitis (LCMV) particles [145].

Using solely deionized water for disruption showed higher infectivity yield compared to a combined approach with sonication and FT-cycles, which presumably impaired viral integrity. On the other hand, the use of a low ionic buffer to establish hypotonic conditions was insufficient to release VPs. Moreover, the additional use of sonication and FT-cycles did not enhance infectivity and may have further reduced it. Kong et al. observed diverging results using double distilled water to lyse two different cell types under hypotonic conditions. The recovery of infectious avian metapneumovirus (aMPV) by water lysis compared to FT-cycles was only beneficial for an avian cell line (TT-1) and not for Vero cells [146]. Env. VPs seem to mechanically withstand the osmotic pressure in hypotonic environments, as shown for influenza particles, which only react by swelling but without loss of activity [147]. Despite these advantages, hypotonic cell disruption is rarely used, possibly due to the buffer exchange required and, thus, the lack of scalability. Moreover, hypotonic environments failed to efficiently release VPs from cells and cell debris attachment, supposedly due to high prevailing electrostatic interaction in low ionic environments [148].

Chemical lysis is the method of choice for non-env. VP production due to ease of use and scalability. In the case of env. VPs, most detergents impair the VP due to the exposed membrane proteins [149]. Furthermore, lysis chemicals might pose a safety risk in vivo, and their removal must be considered during purification [150].

Overall, cell disruption methods can increase infectious VP titers accessible for further purification but also release cell-derived impurities. It increases the burden for the purification process, e.g., impurities can bind to chromatographic resins, thus decreasing binding capacity and the probability of filter fouling as filtration steps increase. The application of cell disruption and increased purification burden needs to be justified by increased VP yields at sufficient purity.

### 4.3. Viral Release

Even after cellular egress of VPs, viral particles can still be attached to the cell surface. For vaccinia, these cell-associated VPs are believed to drive cell-to-cell infections in combination with an actin tail propulsion [136]. Cell-associated VPs can be released by the addition of release agents. The use of heparin and dextran sulfate was shown to dissociate VPs from cells and cell debris through electrostatic interaction with viral envelope proteins [148]. Increased titers were also observed for VSV after the addition of salt or dextran sulfate [124,151]. An electrostatic interaction hindering the release of VPs into the culture supernatant was presumed.

### 4.4. Nuclease

Nucleases are used to degrade hcDNA and reduce its associated viscosity in the harvest. The high cost of these enzymes and their narrow optimal working window require targeted placement within the process. Process conditions, such as pH, temperature, incubation time, ionic strength, nuclease type, and nuclease concentration, have a high influence on nuclease efficacy and can easily be optimized by a DoE approach [152]. Cell disruption typically releases high amounts of hcDNA, and thus, a subsequent nuclease step is commonly implemented, though the large working volume at the harvest stage necessitates a high amount of enzyme, increasing the costs for this digest step.

In a VSV production process, the placement of a nuclease step at various purification stages was tested, and the nuclease step immediately after the harvest stage facilitated the best overall VP recovery [153]. In addition to measuring the reduction in hcDNA, infectious titers need to be monitored, as elevated temperatures and incubation times can reduce infectivity [154]. In some cases, the nuclease step had an additional positive influence on the clarification recovery, as observed for an orf virus production [152]. Filterability was not only improved due to viscosity reduction but also due to a hypothesized dispersion of large aggregates of VPs bound together by DNA chains. In a study by Mayer et al., the nuclease step improved the recovery of a subsequent affinity chromatography step without reported negative impact [155].

Destabilization of chromatin structures by salt addition has been reported [156]. The tight packing of DNA with histones in chromatin shields DNA chains from nuclease digestion and the application of salt-tolerant nucleases in increased salt conditions was shown to improve DNA removal [157]. In another report, Vincent et al. assumed negative impacts of chromatin/VP complexes on a chromatographic purification step [158]. The positively charged chromatin reduced the binding capacity of a cation-exchange chromatography (CEX) column through binding competition. Furthermore, the binding strength of VPs was increased due to chromatin/VP complexation, presumed due to the co-elution of both at high salt conditions [158].

## 5. Centrifugation Methods

### 5.1. Low-Speed Centrifugation: Clarification

The clarification step aims to remove cells and other insoluble large impurities, such as cell debris, from the cell culture harvest. Differential centrifugation applied at low speeds (<10,000× *g*) is used to pellet large, insoluble components of the cell culture harvest at mild process conditions. Excellent recoveries have been reported for the centrifugation clarification step [159], and in the case of env. VPs, clarification by low-speed centrifugation alone or in combination with a subsequent filtration is commonly used at lab scale [153,160,161,162,163,164]. Centrifugation increases filtration capacity by reducing membrane fouling when performed prior to a filtration step. Sviben et al. compared low-speed centrifugation at 3000× *g* and a 0.45 µm polyvinylidene fluoride (PVDF) filtration for the clarification of mumps and measles VPs. The authors showed higher infectious step recoveries for both VPs for clarification by filtration and observed a reduction in particle size after centrifugation. Shearing in the centrifuge was assumed to reduce the recovery, which is coherent with VPs being sensitive to vortexing observed in the same study [28]. Systematic evaluations of centrifugation properties and its impact on infectious titers are rarely reported. One reason is the difficulty of scaling centrifugation steps due to the lack of transferability across scales in combination with high initial and maintenance costs [159]. Hence, filtration steps are preferred if process scale-up is intended.

### 5.2. Differential Centrifugation: Concentration and Partial Purification

Differential centrifugation is used for the concentration of VPs, which leads to the pelleting of particles under ultracentrifugation force, usually greater than 20,000× *g*. VPs, but also impurities, end up at high concentrations in the pellet, which is afterward resuspended. The force impact on VPs and overcompaction within the pellet was shown to reduce viral infectivity of env. VPs in inverse proportion to centrifugal time and force [165,166,167]. The co-precipitation of impurities (e.g., cell debris, proteins) can lead to neuro-inflammatory responses in vivo, and thus, further purification is required [168]. A high-density cushion, such as a sucrose cushion (SC), can be used to retain smaller impurities while pelleting the VPs to reach partial purification [169,170]. However, chromatographic steps, in comparison, show higher recoveries of infectious particles (for an orf process [15]) and lower amounts of contaminants (for a VSV process [162]).

### 5.3. Density Gradient Centrifugation: Concentration and Purification

Density gradients leverage differences in sedimentation velocities (rate-zonal centrifugation) or in buoyancy (isopycnic centrifugation) to separate particles. The differences are established by high-density media, e.g., sucrose, CsCl, iodixanol, or others. In-depth information can be found in the book Nanoseparation Using Density Gradient Ultracentrifugation, chapter 2 [171]. Depending on the centrifugation conditions and processing step, density gradients are used for VP concentration and/or separation from cells, large cell debris, as well as impurities that are smaller in size. Separation of VPs from EVs and microvesicles has been reported for herpesviruses [49] and HIV [52]. Still, due to the heterogeneity of EVs and their similarities in density and size with env. VPs, a complete separation is not feasible [51].

A high resolution is established in density gradients via long centrifugation time and high centrifugation forces. Both increase the induced mechanical stress on VPs, potentially resulting in the loss of surface structures and, thus, infectivity loss, reported to be up to 99% [172,173]. A high sucrose concentration increases the osmotic pressure [174] in solution, which impairs cell cultures and living organisms when administered intravenously. A 20% sucrose solution resulted in an intense diuresis in rabbits [175]. Lengthy wash steps, such as dilution and pelleting through centrifugation, are applied to reduce sucrose before in vitro or in vivo applications [176].

CsCl, compared to sucrose as a medium, has advantages due to a lower viscosity and faster centrifugation, but both are hyper-osmotic in the concentration range applied. The sudden change in osmotic pressure can impact cell cultures, as well as the viral envelope’s integrity, thus reducing safety and infectivity [147]. Still, protocols of the last decade for the purification of preclinical orf and NDV material include sucrose and iodixanol density gradients [139,177]. In addition to preparative applications, analytical methods exploit the high separation resolution in isopycnic applications to analyze density populations [178] or conduct subsequent proteomics [179].

### 5.4. Centrifugation Summary

Centrifugation methods for concentration and partial purification can induce mechanical stress that leads to lower recovery of inf. VP titers. Scale-up techniques are limited, and thus, industrial preparative processes rely on other methods, as detailed in the following sections [180]. However, density gradient centrifugation techniques are still used ubiquitously in original lab-scale processes and present advantages due to their fast process development and simple application with the possibility for buffer exchange. Especially in virology research labs, these methods are commonly used, even though their purification capabilities and scalability are limited, and their negative effects on viral integrity is known.

## 6. Filtration Methods

Filtration methods are ubiquitously performed in VP bioprocessing. There are filtration membranes of various materials with a range of pore sizes employed in different designs and flow patterns suitable for each specific application. Clarification by filtration uses the steric retention of cells and large particulates from the harvest material, thereby reducing turbidity and enhancing subsequent processability. UF/DF utilizes membranes to retain VPs based on size, effectively concentrating VPs and removing smaller, undesirable impurities and soluble buffer components. Sterile filtration at the end of bioprocesses employs a microfilter to ensure sterility of the final product, but challenges exist for VPs larger than the typical filter cutoff of 0.2 µm.

Env. VPs are prone to adsorption on the filter membrane due to their lipid membrane facilitating unspecific binding primarily by electrostatic or hydrophobic interactions. Unspecific binding was shown to significantly decrease overall yield [181,182] and might result in early membrane fouling, possibly stopping membrane flux. Screening of different filter membrane materials must be performed early on to identify a suitable material for the particular VP of interest.

Charged membranes and filter aids are designed to reduce impurity content, typically applied as depth filters in clarification. They have been shown to be unsuitable for VPs because the strong electrostatic binding reduces VP yield [152,154]. For lentivirus (LV) purification, however, the use of diatomaceous earth (DE) as a filter aid simplified the clarification [183]. DE increased the filtration capacity and purity by prolonging the processing time until clogging and cake formation. Even though this one-step filtration reduced inf. VP yield by 35% compared to the alternative two-step clarification, it was regarded beneficial by the authors due to faster processing, safer VP handling, and improved robustness [183].

Filter membranes of different materials are available, such as PVDF, polyethersulfone (PES), polypropylene (PP), glass fibers (GF), nylon (NYL), and cellulose-based membranes like cellulose acetate (CA), cellulose esters (CE), nitrocellulose (NC), and regenerated cellulose (RC). Suitability tests for env. VPs are scarcely reported, and a thorough screening including the influence of pore sizes and feed parameters is lacking. The application of filter devices incorporating multiple filters with different materials as coarse prefilters hinders a distinct material evaluation [184].

A recent study by Labisch et al. for the purification of LVs screened different materials and pore sizes of filters in three different devices: centrifugation filters, stirred cells, and crossflow cassettes [185]. Interestingly, the authors found the overall best-performing filters in terms of inf. VP recovery and impurity removal to have different pore size specifications: reinforced 100 kDa PES, 300 kDa cellulose-based filter. The reinforced PES membrane outperformed other membranes across all devices, also in comparison to non-reinforced PES membranes. This was contributed to tighter pores due to polymer fleece reinforcements within the PES membrane, resulting in optimal retention of LV particles while effectively passing small impurities. Large differences in inf. VP recovery were found between the individual filter devices, even with the same membranes. This emphasizes the impact of device design and flow patterns on concentration polarization, filter fouling, and separation performance [185].

For a primary clarification of influenza VLPs, Carvalho et al. observed complete recovery of HA activity when applying different filter materials with pores sizes between 0.55 µm and 10 µm (PP, PVDF, and depth filters with material combinations of cellulose, PP, glass fiber, and DE filter aid) (Carvalho et al., 2019 [186]). In a second clarification step utilizing pore sizes of 0.2 to 1.2 µm, most materials (CE, PES, and mixed material depth filters) showed complete recoveries, whereas PVDF and PP showed reduced functional recoveries. Only the PES filter additionally reduced residual, and in this case contaminating, baculovirus (BV) particles, most likely due to steric hindrance.

Especially for later purification stages with reduced protein impurities or no excipients, which prevent unspecific interaction, the material choice has a substantial impact [181,187]. Pre-coating of membranes using proteins can improve recoveries [182,188] but also introduces impurities to the process. On the other hand, residual hcPs at levels of about 25 µg/mL have been shown to negatively impact the sterile filtration step for VSV on PVDF and PES membranes through accelerated filter fouling [189].

It is worth noting that PVDF belongs to the category of per- and polyfluoroalkyl substances (PFAS) for which the European Union plans a ban due to health and environmental hazards [190].

### 6.1. Normal Flow Filtration (NFF)

In NFF, also known as dead-end or direct flow filtration, the fluid is passed perpendicularly through the filter membrane, trapping the larger particulates on the filter surface. Common applications for envelope VPs involve clarification and sterile filtration. At the lab scale, centrifugal NFF devices are also applied in sample preparation for volume reduction [15,28,185].

#### 6.1.1. NFF for Clarification

Clarification by filtration aims to separate large contaminants in the retentate while VPs are collected in the filtrate. A large variety of filter materials and modalities exist, and the optimal devices need to be chosen based on the VP and feed properties, as well as purification requirements. Due to the broad size range of particulates to be depleted in clarification, multiple subsequent filters with degressive pore sizes (filter train) or a combination with other clarification methods are used [129,154,160,162]. A filter train increases the capacity of the latter filters by pre-filtration steps for large particles. At lab scale, membrane areas can easily be enlarged; however, at large scale, this might lead to practical and economic difficulties [152]; thus, filtration trains are preferred [191]. In the case of prior cell lysis, the burden on the clarification step is increased, usually necessitating a pre-filtration. In a protocol for a lab-scale orf virus production, the exchange of the pre-filter after cell lysis is recommended due to fast filter fouling, highlighting the high burden on the subsequent clarification step [139].

As each VP and purification process has its own requirements and specific feed properties, finding the optimal inert filter material and filter pore size requires extensive screening. Small screenings of filter materials might not result in a satisfactory balance of VP recovery and impurity clearance. This was observed for replication-defective HSV particles, leaving room for optimization after four materials (NC, NYL, CA, PES) were tested [167]. Modern high-throughput (HT) screening devices such as the Ambr crossflow (Sartorius, Göttingen, Germany) facilitate this effort, as shown by Pagallies for an orf virus clarification [152]. Filter properties, as well as nuclease treatment and TOH, are significant factors influencing the recovery of the clarification step. After HT screening and optimization, a recovery of up to 80% was reached for a combination of two PP filters, while turbidity was reduced to a third of the initial level. Additionally, a prior nuclease step improved recovery, presumably due to the breakdown of DNA-VP complexes, as discussed in Section 4.4. Other reports showed a similar breakdown effect by the addition of salt [129] or the introduction of a homogenization step [192], whereas the latter led to increased impurity content in the filtrate.

#### 6.1.2. NFF for Sterile Filtration

Conventional final filtration in biopharmaceutical processes consists of a sterile filtration step to fulfill regulatory requirements to deplete potential bacterial contaminations. The same microfiltration step of 0.22 µm has been applied for various env. VPs, though, in general, high product losses were reported for PES and PVDF membranes [189]. Transfiguracion et al. screened filter membranes and the influence of buffer matrix for the final filtration of BV particles [193]. Unspecific binding was presumed to reduce recovery to approx. 20% for a PES membrane. The addition of salt did not increase recoveries, in contrast to the well working improvement by salt addition for the prior SEC step. The tested polysulfone (PS) membrane yielded a maximum inf. VP recovery of 79%. Again, salt addition did not improve recovery but led to VP inactivation [193]. In another screening by Shoaebargh et al., the influence of filter material morphologies was tested for the filtration of VSV particles [184]. Total particle recoveries were generally low (<25%), and no correlation to membrane morphologies or the incorporation of pre-filters could be taken. However, utilization of two-layered filters delayed transmembrane pressure (TMP) increase and aided the filterability. This was attributed to filter blockage by VP aggregates [184], in line with the findings of a recent study for a vaccine VP [194]. On the contrary, Fernandes et al. had no issue with the final filtration of VSV particles [154]. The sterile filtration utilizing a PES membrane achieved full reported recovery of inf. VPs. It is unclear whether the differences arose from different VP formulations or the higher purification state in the study by Fernandes [154]. Considering protein impurities, Wright et al. observed the impact of residual hcPs in accelerating membrane fouling. An effect of hcP resulting in filter blockage or the mediation of VP attachment to the membrane was presumed [189]. However, as the last step in the purification step, hcP levels should be sufficiently low to prevent these effects.

Larger env. VPs such as poxvirus and vaccinia viruses exceed the exclusion limit of 0.22 µm sterile filters. Larger filter pore sizes such as 0.45 µm or 0.65 µm have lower bacterial retention capacity, and their application, if utilized, must be justified. As stated in regulatory guidelines [195], an aseptic process may become necessary, but this requires much higher technical efforts.

Sterile filtration of VPs is a crucial step that can limit overall process recoveries tremendously. The step performance is impacted by variabilities in the purification process. Screening for the optimal membrane material for filtration of VPs in its formulation must be performed, and the amount of VP aggregation before and after filtration, as well as the influence of residual proteins, should be controlled.

### 6.2. Tangential Flow Filtration (TFF)

TFF is an essential technique in the bioprocessing of env. VPs. It operates by allowing a feed solution to flow tangentially across a selective membrane, reducing the build-up of solids on the filter and prolonging the operational life of the filter medium. For harvest clarification, VPs are filtered out in the permeate while the cell culture and large debris remain in the retentate. This can be implemented in a perfusion culture setup as well as a batch setup. The main application of TFF is the retention of VPs enabling concentration and buffer exchange. It is readily scalable, rendering it very interesting for industrial applications [196].

TFF applications have been shown to successfully concentrate shear-sensitive measles VPs using the lowest possible TMP [187]. Depending on the application and feed properties, higher flow rates in the retentate loop may be required to reduce membrane fouling and speed up processing times. Peristaltic pumps, commonly used for TFF systems, have been correlated with reduced recoveries of VPs [38,196] and attributed with increased shear rates [197]. ATF utilizing diaphragm pumps as well as centrifugal pumps designed for low shear rates have been shown to prevent membrane fouling as well as improving recovery for shear-sensitive particles [197]. It is worth noting that specific glycoproteins on the envelope of VPs can increase viral stability, as shown for HIV VLPs [90]. As discussed in Section 2.2.2, env. VPs can be engineered for stability by design to sustain mechanical forces during the purification process.

Hollow fibers and flat sheet cassettes are commonly used for env. VPs or VLPs [187,198,199], although no comparative study between these devices was identified at this time. Differences in surface area per volume, flow distribution, scalability, and ease of use are apparent and need to be evaluated for the specific use case. Nonetheless, hollow fibers are presumed to induce less shearing due to larger internal channels [200]. Performance and recovery of inf. VPs seem to be largely dependent on filter material and pore sizes and less dependent on the specific device used.

#### 6.2.1. TFF for Clarification

TFF microfiltration can be used to prevent early membrane fouling in clarification without increasing membrane area or filtration steps. For example, in the production of HIV VLPs, a TFF microfiltration step using a 0.45 µm filter was used for clarification prior to a second TFF UF step with a 500 kDa cutoff membrane [199]. An overview of the advantages and disadvantages of filtration methods is provided by Besnard et al. [159]. Generally, TFF is more laborious compared to NFF and is only beneficial for high volumetric feeds. Thus, it is mainly used for lab-scale harvest if a continuous or repeated harvest is developed, see Section 4.1. At large scale, TFF applications have not been reported for clarification purposes, although filtration modules for scale-up are commercially available for pore sizes 0.1 µm and larger. The effect of shear stress in TFF systems for VPs during the processing of large volumes must be considered, although this may be controlled with an optimized flow rate.

#### 6.2.2. TFF for Buffer Exchange and Concentration

TFF is primarily applied as UF/DF. It is a useful method for processing feed concentration and buffer exchange, for example, at the end of a production process for formulation and adjusting the VP concentration to reach specified titer ranges. For env. VPs or VLPs particle recovery rates of up to 100% have been reported [198]. However, the impact on replication-competent VPs has not been evaluated in many studies. Fernandes et al. used a hollow fiber setup for UF/DF of VSV particles. After 6 times DF and 4 times concentration, the authors reached an inf. VP recovery of about 50%. No impact of shearing was observed as an increase in flow rate achieved similar inf. VP recoveries but rounded, non-native particles have been observed in transmission electron microscopy (TEM) analysis [154]. Another study reported the regular occurrence of such rounded particles in VSV bioprocessing [83]. Loewe et al. screened different TFF membranes for their UF/DF application for measles VPs [187]. They reported no impact of filter material on retention of VPs and that a 15-times concentration could be achieved for three different membranes; however, no inf. VP recovery values were reported.

### 6.3. Filtration Summary

Filtration is essential in VP bioprocessing for clarification, concentration, buffer exchange, and, in the case of small VPs, sterile filtration. However, infectivity loss and also membrane fouling are challenges to be addressed with enveloped VPs. Optimization of a filtration step is crucial but may require extensive screening of filter materials (e.g., PVDF, PES, PP). Charged membranes and filter aids are generally unsuitable despite some success with DE for lentivirus clarification.

Filter design and flow patterns significantly impact VP recovery, while material selection becomes especially important in later, more purified stages to minimize unspecific adsorption. NFF is favored for clarification, and filter trains starting with coarse filters are especially useful when the feed is crude harvest. TFF is suitable for concentration and buffer exchange, though some pumps (e.g., peristaltic) and a non-optimized setup can damage shear-sensitive VPs. Sterile filtration is not always possible for the bioprocessing of larger VPs, necessitating aseptic conditions for the entire process.

Optimizing filtration of enveloped VPs demands extensive screening of materials, devices, and parameters to address binding, fouling, and product loss. Tailored strategies are essential for effective clarification, robust and high VP recovery, and a sterile drug substance.

## 7. Chromatography Methods

Chromatography is a conventional purification method widely used for VPs. SEC, as well as ligand-based modalities such as IEX and HIC, are commonly used. Moreover, specific affinity ligands were recently developed for VP application. Stationary supports that facilitate high mass transfer and high flow rate are especially beneficial for VP purification. The impact and suitability of relevant modalities and stationary phases are discussed in the next subsections, together with the evaluation of relevant published studies. Chromatography in bind-and-elute (B&E) mode is favored as an initial capture step to process large volumes with typically low VP concentrations at the harvest stage. Chromatography in FT mode is designed to retain impurities and is thus typically used as a polishing step to deplete residual impurities.

### 7.1. Chromatographic Modalities

#### 7.1.1. Ion Exchange Chromatography (IEX)

IEX might be used as capture for VP purification in B&E-mode after a clarification step as well as a polishing step to further deplete impurities. It operates based on charge, which is dependent on the isoelectric point (IEP) and surrounding pH. The IEP of env. VPs is not easily predictable due to the high variability of envelope lipid and glycoprotein composition. Published experimentally determined IEPs for env. VPs are rare, and multiple literature values might not match due to a high dependency on the upstream [201].

Env. VPs carry an overall negative charge at neutral pH, which points to the use of anion-exchange chromatography (AEX). Several publications show the successful application of AEX chromatography with infectious step recoveries of up to 86%, including NDV and influenza on AEX monoliths [19,202] and VSV pseudovirus on an AEX membrane [154]. However, the net charge itself is not sufficient to determine suitable process conditions for the IEX step. Measles VPs have been shown to form such a strong electrostatic binding to AEX resins that it is not possible to recover infectious VPs [45,203]. Instead, a CEX column was utilized by Eckhardt et al., indicating sufficient positive patches for binding on the VPs. Additionally, Gautam et al. used a CEX membrane adsorber for the purification of VSV particles [162].

Impurity content and properties, as well as VP properties, are cell culture-dependent and variable [158,163], and thus challenge the robustness of an IEX step and its suitability as a platform process step. Impurities carrying similar charge properties are co-purified and reduce the binding capacity for VPs. Multi-point binding of VPs, further discussed in Section 7.1.5, is established through ligands conjugated on linker polymers, as in tentacle resins. This increases binding strength to the resin and sets it further apart from weakly bound smaller impurities. By modulating the salt concentration during loading, ‘interference chromatography’ [204] aims to exploit the ‘sweet spot’ in which VPs bind, but smaller impurities with fewer surface charges do not. This method is presumed to be especially successful for high ligand-density IEX adsorbers and increases binding capacity as well as separation performance. Fernandes et al. showcased the beneficial utilization of citrate for the purification of VSV particles on an AEX column, increasing the dynamic binding capacity (DBC) and purification performance [154].

IEX chromatography performance depends on loading parameters such as pH and salt concentration. The capture of VPs directly from clarified harvest without adjustments requires a robust unit operation that can withstand expected feed variabilities. Rogerson et al. used a HeLa cell culture to produce NDV VPs and evaluated the influence of pH and conductivity for the AEX capture step [19]. In the range of pH 7 to 7.6 and loading conductivity of 8 to 17 mS/cm, their robustness study showed a constant recovery of infectious particles of 25% to 33% and an impurity reduction of above 53% for hcDNA and 89% for hcP was reached. However, in an upscaled application, an inf. VP recovery of 37% with a StD of 14% (incl. assay variability, *n* = 5) was realized. Harvest composition variabilities due to the upstream were assumed to be responsible for the increased variability.

IEX for the polishing step in FT mode can be performed after a concentration step. The stationary phase is functionalized ideally with ligands only accessible to small impurities. Restricted access media (RAM) is specifically designed for this application and discussed in Section 7.2.7, including polishing chromatography.

#### 7.1.2. Affinity Chromatography

Affinity chromatography is the preferred capture method in biopharmaceutical processes due to its high specificity and, therefore, high purification capability. As a prerequisite, a suitable ligand/target combination must be identified while considering purification capabilities and economic factors. Currently, applications of affinity chromatography methods are found mainly for vaccines and VLPs, for which Lothert et al. give an extensive overview [205]. Transferable knowledge for this technique to replication-competent env. VPs is summarized here, together with the evaluation of relevant published studies.

Heparan sulfate on cell surfaces assists in the attachment of VPs. The specific affinity of VPs to heparan sulfate and its analog heparin have been exploited in affinity columns for the capture of VPs. Nasimuzzaman et al. showed a strong binding of BV particles to a heparin column, which requires high salt concentrations for virus elution [206]. Due to an immediate stabilization by dilution of eluted particles, inf. VP recoveries of up to 85% were reached [206]. Due to the animal origin of heparan sulfate and heparin and the high costs for recombinant alternatives, heparin-mimicking sulfated polysaccharides have been developed. Sulfated cellulose and dextran have been successfully utilized for enveloped virus vaccines such as MVA [207] and influenza [208] but showed only very low inf. VP recovery for measles VPs in comparison to a heparin purification [155].

The development of new ligand/target combinations for env. VPs is an ongoing process. Engineering VPs to include targets on the surface, such as biotin-mimicking tags to bind to streptavidin [209] or histidine tags for metal affinity [210], were successfully implemented for enveloped VLPs and vaccines but come with limitations for applicability for replication-competent viruses. The impact of such tags on replication cycles and in vivo effects is unknown, and leachable metal ions lead to virus inactivation [211].

More recent studies applied in silico modeling and experimental screening setups to identify ligands with high specificity. For instance, LVs with VSV-G glycoprotein were successfully targeted [160]. Promising results were reported in other studies that used phage display for LV particles [212] and coronavirus purification [213]. However, the transferability of customized ligands to capture replication-competent VPs remains outstanding. The advantages of high specificity must be balanced with the high development efforts required, and this strategy may only be beneficial for widely used virus platforms with conserved glycoproteins. The question of cost/benefit, column production cost, reliability in supply and quality, and column reusability for potentially expensive ligands remains.

#### 7.1.3. Hydrophobic Interaction Chromatography (HIC)

HIC has been successfully used as a capture step in the purification of env. VPs. Sviben et al. showed infectious recoveries for mumps and measles VPs of over 60% [45]. High titers could be achieved while hcDNA was depleted. For vaccinia particles, an inf. VP recovery of above 50% was reached, while hcDNA was reduced by 99% [158]. In both studies, hcDNA was depleted without prior nuclease treatment. However, when HIC was applied as a polishing step for MVA, it was not sufficient to deplete the residual hcDNA after an affinity capture step [214]. It is noteworthy that only double-stranded nucleic acids flow through the resin due to their hydrophobic patches being shielded by the double structure, unlike single-stranded nucleic acids, in which hydrophobic patches are exposed [215].

There are some notable disadvantages to HIC. A high salt concentration is required for the binding of particles. Ammonium sulfate is typically used for the binding of VPs, but at high concentrations, it has been found to precipitate and inactivate VPs [45,158,216]. Vicente et al. substituted ammonium sulfate with NaCl to prevent aggregation and resolve filterability issues of vaccinia VPs. The impact of salt concentration on the flowthrough of hcDNA was found to be minimal [158,214]. In all applications, a balance between the salt concentration required for VP binding and the detrimental effect on the viral integrity must be determined. HIC is, thus, best-suitable for VPs with a high salt tolerance.

#### 7.1.4. Steric Exclusion Chromatography (SXC)

SXC has emerged as a promising technique for the purification of VPs, leveraging specific properties of polyethylene glycol (PEG). The method is based on steric exclusion effects, where PEG molecules induce a crowding effect that facilitates the capture of large targets on a hydrophilic stationary phase without direct binding [217]. SXC is particularly advantageous for the purification of large, labile biomolecules due to its gentle nature and the absence of direct chemical interactions, which helps maintain the structural integrity of sensitive viral components [218]. Furthermore, the addition of PEG to the load material is presumed to shield VPs from shear stress [219]. The application of SXC for env. VP purification has been shown in various studies, the majority being vaccine or VLP applications, including BV [220], influenza [221], Hepatitis C [222], and LV [219].

Two studies are available that capture replication-competent orf VPs using SXC, achieving inf. VP recoveries of 85% [15] and over 90% [70]. Protein depletion is efficient, with over 98% protein reduction in both studies. For hcDNA reduction, a prior nuclease step was used to increase its size difference to VP, and thereby reductions of about 80% were achieved. Even though the inf. VP recovery was much higher for SXC compared to SC purification, the eluted concentration of orf was in the range of 10^6^ IU/mL, and thus three log-steps lower compared to SC [15]. Eilts et al. analyzed the VP quality after SXC and observed a monodisperse size distribution of VPs and minimal morphological changes in TEM, indicating a good VP quality [15]. Lothert et al. further evaluated the method for two different virus genotypes. Pressure issues arose for one genotype during loading, presumably due to the genotype’s surface properties and resulting aggregation of VPs. In a low-loading setup (<DBC_10%_) of SXC, similar performances for both genotypes were observed, showing the method’s robustness for virus strain variations. No relevant aggregation levels were observed after SXC purification [70]. However, both studies presumed residual PEG molecules after the process despite a second purification step. PEG removal and monitoring after the SXC step is not yet sufficiently reported and poses a safety concern due to the rare prevalence of PEG allergies [218].

SXC purification performance and DBC, as well as the extent of VP aggregation and resulting pressure increases, are dependent on process parameters. PEG concentration and molecule size and the feed matrix in terms of buffer composition can be optimized. High recoveries were shown to be achievable for different viral strains, whereas the infectious recoveries for labile VPs such as influenza and measles are still to be evaluated. Additionally, PEG is introduced at high levels, which must be treated as a process impurity at subsequent purification stages. Additionally, as the collection of VPs on the initial membrane surface leads to pressure issues and potentially reduced binding capacities, there remain challenges in membrane design that must be addressed to facilitate the scaling-up of this method to production scale [223].

#### 7.1.5. Surface Functionalization

The manner in which ligands are presented influences both their ability to bind (binding capacity) and the strength of their interactions (binding strength). Polymers can be grafted on a stationary support to act as ligand linkers, extending the reach of ligands and increasing the overall ligand density. These flexible linker polymers are termed tentacles. A multi-point binding principle is assumed to be facilitated through the flexibility of linker polymers and increased ligand density. The multiple binding points increase the overall binding strength and make the interaction robust in the case of IEX against changes in salt concentrations. Increased salt concentrations in the feed prevent the binding of impurities to the column, increasing the separation efficiency and capacity for VPs [204,224]. Eckhardt et al. showcased the higher performance of Eshmuno CPX (Merck, Darmstadt, Germany), a bead-based resin using linker polymers, in a screening of IEX bead-based resins for the purification of measles VPs [203]. Aguilar et al. showed the sufficient binding of enveloped VLPs on the exterior surface of linker polymer bead resin [225]. Moreover, the use of linker polymer resins is also highlighted in the literature for their efficacy in viral clearance [226].

On the contrary, reports also show that ligands at high density can result in a binding strength that negatively impacts the recovery and infectivity of VPs. Turnbull et al. showed a time- and ligand-density-dependent yield reduction of non-env. VPs for ligands directly grafted on an AEX fiber resin [227]. A high ligand density in combination with an increased polymer layer thickness was also shown to reduce the infectivity of eluted env. VPs and lead to co-purification of impurities [224,228]. Pamenter et al. observed time-dependent two-step adsorption of LV particles on a strong AEX membrane adsorber [229]. The authors hypothesized a conformational change as the second binding step, resulting in a strong multi-point binding. Strongly bound VPs could only be eluted during the cleaning-in-place (CIP) step. A kinetic approximation resulted in a halving time for the second step of approximately 15 min. A more rigid VP is presumed to withstand the induced conformational change [229].

In ligand density screenings for enveloped and non-env. VPs, the lowest tested density yielded the highest recovered infectious titers [224,228]. Low ligand densities and a shallow polymer layer also reduce ligands that are accessible only to smaller impurities and, thus, decrease the co-purification of such, as observed by Vicente et al. [228].

Parameters such as polymer layer thickness, polymer density, and ligand density remain to be optimized specific to each VP application. This is not feasible within a virus process development. Moreover, customized stationary phases carry a high cost for later upscaling. It is also worth noting that grafted polymer layers reduce the pore or channel diameters, which might result in increased backpressure during loading, prolonging the processing time.

#### 7.1.6. Size Exclusion Chromatography (SEC)

Preparative SEC can be used to separate VPs from smaller, soluble impurities. SEC resins exclude VPs from the resin pores while impurities can enter, resulting in VPs eluting in the void volume and impurities at later fractions. However, it cannot effectively separate impurities near the size of VPs. A buffer exchange can be implemented, which makes this step suitable for polishing. As low flow rates and low feed volumes are required for SEC, a prior concentration step might be needed. SEC steps are characterized by low productivity, lack of scalability, and inevitable dilution [230].

The exclusion of the large VPs, and thus, good separation from impurities, facilitated an overloading of BV particles on a Sepharose SEC column (Cytiva, Marlborough, MT, USA) with still good purification performance [193]. With this approach, the low productivity of SEC was improved by Transfiguracion et al. The SEC nonetheless took 85 min using a 69 mL column and achieved a step recovery of 73% total particles and 64% inf. VPs [193]. Kröber et al. addressed the low productivity of SEC columns by utilizing a simulated moving bed (SMB) approach and coupling multiple columns in a continuous process for the purification of influenza particles [231]. The authors achieved a maximum productivity increase of factor 3.8; however, a scale-up would require longer processing times, thus reducing productivity [231]. An early publication for the purification of coronavirus particles reported superior capabilities of SEC purification compared to overnight SC purification [216]. However, no time benefit was achieved by the utilization of the 466 mL column requiring over 7.5 h for one column exchange.

One advantage of SEC is its ability to facilitate buffer exchange and desalting. This feature allows the use of a favorable buffer environment, which ensures virus integrity during the process. However, it is important to carefully select buffer conditions to prevent any unspecific interactions of VPs which could negatively impact the purification as well as the viral particle integrity. Transfiguracion et al. highlighted the importance of buffer screening and optimization due to the fact that three out of four screened buffers had an initial infectious recovery of below 10% for BV particles [193]. After buffer and salt concentration optimization, a maximum of 97% infectious yield could be reached.

SEC has been observed to exert shear stress on large molecules, a phenomenon that has been particularly noted in polymers and HPLC applications [232]. A study for preparative SEC purification reported significant activity reduction for a non-enveloped and inactivated VP in the foot-and-mouth disease virus (FMDV) [233]. Shearing of VPs within pores has been found to cause alterations in the secondary and tertiary peptide structures on the surface of viruses. However, the negative impacts on VP stability and immunogenicity could be overcome by stabilizing buffer components [233]. In the case of env. VPs, the usually larger particle sizes exclude VPs from entering pores, thus reducing the observed impact. The hydraulic diameter, a value characterizing the flow pattern in the column, has been found to be inversely proportional to the shear rate. Larger resin beads and lower flow rates thus reduce shearing [234].

### 7.2. Chromatographic Stationary Phases

**Figure 2:**
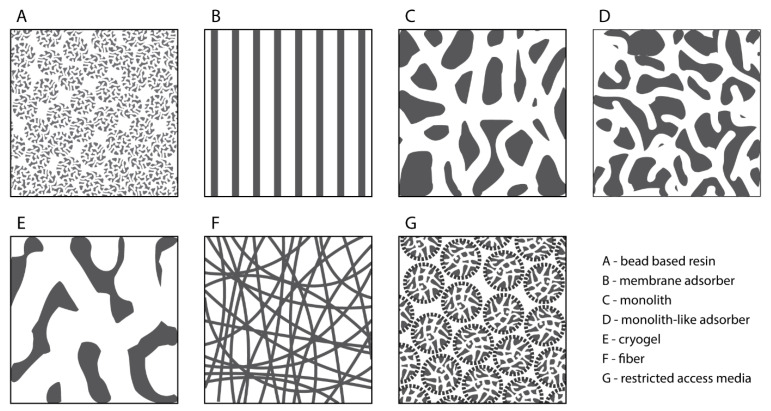
Illustrated schemes of chromatography stationary phases applied for the purification of virus particles. Illustrations not to scale.

Various conventional, as well as novel stationary phases have been applied for the purification of virus particles. Illustrated schematics of subsequently discussed stationary phases are shown in Figure 2.

#### 7.2.1. Bead-Based Resins

Conventional chromatography resins typically used for antibody purification are based on porous and functionalized beads. These beads have a highly porous internal volume to increase the functionalized surface area. The internal bead volume is not accessible to enveloped viruses due to steric hindrance based on their large size. Additionally, the low diffusional movement of VPs leads to a high mass transfer resistance. Consequently, the binding capacity of bead-based resins for large VPs is primarily dependent on the outer surface area of the beads, which in turn is influenced by the size of the beads [235]. The size of the beads also has a direct impact on backpressure and shear rates and, thus, the applicable flow rate. Specifically, smaller beads result in an increased binding capacity, but also increased backpressure. This necessitates a reduction in flow rate, rendering this setup unsuitable for a capture step in B&E-mode in which high volumetric feeds need to be processed. In contrast, larger beads induce low shear rates and have a reduced binding capacity of VPs while sustaining a high binding capacity for impurities. This makes bead-based columns generally more suitable for polishing applications in FT mode instead of B&E capture applications. Specifically optimized resins for VP polishing applications are RAMs, which are discussed in Section 7.2.7.

The application of VP binding on bead-based resins is explored in several publications. In one study, Rogerson et al. conducted a screening of AEX resins, evaluating the DBC specifically for NDV VPs of size 100 to 500 nm [19]. Their findings revealed a 10-fold higher DBC when using a Poros AEX resin (Thermo Fisher Scientific, Waltham, MA, USA) in comparison to other resin beads. The authors attributed the difference to the larger pore structure of Poros resin. Poros resins are perfusion resins featuring a bimodal pore size distribution of convective macropores and diffusional micropores. Macropores were examined to be in the size range of 100 to 500 nm by Wu et al. [236]. Contractionary to Rogerson et al., Wu et al. observed that human papillomavirus (HPV) VLPs of 50 and 100 nm in size blocked the macropore structures at the surface of the Poros beads and, thus, the internal bead structures were not accessible [236,237]. It is unknown if the observed differences are attributed to different ligand chemistries or the differences in VP types. Regardless of the contradiction, Rogerson et al. demonstrated an NDV concentration ranging from 25- to 60-fold, alongside a 90% reduction in impurity proteins. Despite the promising results with Poros resins, Rogerson’s research suggests that convective chromatographic media, such as monoliths, may still offer superior performance. The study reported DBCs up to 7 times higher using a monolith [19].

The use of chromatographic beads featuring tentacle resins, such as Eshmuno (Merck, Darmstadt, Germany), has been highlighted in the literature for their efficacy in viral clearance. This was demonstrated in a study by Aguilar et al. in which an enhancement in specificity and binding capacity was observed [225]. This improvement is believed to be due to an increased virus-accessible functionalized surface area on the bead’s exterior and increased binding strength, as discussed in Section 7.1.5. This aligns with a screening of CEX resins conducted by Eckhardt et al. [203] for the purification of oncolytic measles VPs. Despite the size of measles particles (100 to 300 nm) exceeding the pore size of the beads (80 nm [238]), the Eshmuno CPX column still exhibited the best performance in terms of recovery. A notable disparity between the DBC and the static binding capacity (SBC) was observed by Eckhardt et al.; only 2.3% of the SBC could be realized in DBC in regard to inf. VPs. The potential impact of shearing was considered, as higher flow rates were observed to result in lower infectious particle recovery. In spite of that, the total particle count, including inactivated VPs, was not determined, and thus, slow mass transfer rates can be causative as well. However, the lab-scale experiments yielded an infectious recovery of 81%, albeit with a 17% reduction in the infectious particle ratio. Significant decreases in total protein and hcDNA were observed, with removal rates of 98% and 81%, respectively [203].

Flow rate-related inactivation of env. VPs was observed by Ta et al. on a different AEX tentacle resin, the Nuvia HP-Q (Bio-Rad, Hercules, CA, USA) [239]. In this case, shearing was determined to result in the fragmentation of VPs and, thus, inactivation of influenza VPs. A flow rate reduction improved inf. VP recovery to some extent; however, another reduction did not recover further inf. VPs. A structural change due to strong binding was assumed to be partially responsible for the virus inactivation [239]. Pamenter et al. provide a mechanistic explanation for structural changes of env. VPs due to binding to strong AEX on tentacles [229], which is discussed in Section 7.1.5.

#### 7.2.2. Membrane Adsorber

Membrane chromatography features convective channels through the membrane stationary support with the benefit of low backpressure and high flow rates during loading. These attributes are ideal for early high-volumetric purification stages. The fast mass transfer in convective channels without diffusional limitations ensures constant DBCs over a wide range of flow rates. Membranes with additional diffusional pores are available, but due to the size and diffusional limitations of VPs, these membranes are more suited for polishing VPs in FT mode.

The adsorber membrane is manufactured as a flat sheet and is then packed in a suitable housing to provide an inlet and outlet stream, as well as an ideally even flow distribution over the membrane area. The low ratio of bed height-to-frontal area, typical for membranes, challenges device design to keep an even flow distribution over the membrane. This is achieved through void volumes in the housing, which also facilitates unfavored dispersion and local concentration of particles, thus resulting in early breakthrough and low resolution [240]. Device designs used for lab-scale applications, stacked-disc devices, as well as upscaled radial flow devices, were shown to have low resolution [241]. Lateral flow devices, engineered specifically for large-scale applications, address these issues and, thus, yield an overall better performance [241]. Still, flow velocity heterogeneities that prevail may induce shear stress on particles at high flow rates [242]. The membrane adsorber resin itself has good linear scalability, but the different scale performances need to be considered. With the development of mechanistic models, the effects of hydrodynamic dispersion can be decoupled from particle/membrane interaction and can, thus, support scale-up, even across different device designs [243].

A grafted polymer layer on adsorbers can increase the binding capacity for VPs and impurities as well as inactivate VPs, as discussed in Section 7.1.5. Gautam et al. used a CEX membrane adsorber including a grafted polymer for VSV purification [162]. Sufficient impurity reduction for in vivo studies was reached by the membrane adsorber with an hcP dose level of approx. 40 ng/10^9^ TCID_50_. No deformation of particles was observed in TEM analysis, and in vivo studies showed no difference in comparison to a clinical grade process. The question remains if an optimized grafted polymer layer can further reduce the level of co-purified hcPs. Gautam et al. reached a high concentration factor for VPs using the membrane adsorber; however, presumably due to stringent cutting criteria and low peak resolution, the recovery of infectious particles in the elution fraction was only 30%. Furthermore, a six-times dilution had to be introduced to reduce the salt concentration that was required for the CEX elution [162]. Fernandes et al. evaluated two membrane adsorbers with grafted polymer layers (Sartobind Q, Sartorius, Goettingen, Germany and Mustang Q, Cytiva, Marlborough, MT, USA) in an IEX capture chromatography screening for a VSV pseudovirus purification [154]. Initial screening showed higher performance of these membrane adsorbers compared to the bead-based column in terms of inf. VP recovery and impurity reduction. The use of citrate in the feed improved impurity removal and increased binding capacity for VPs on the membrane. In the final process, an inf. VP step recovery of 80% could be reached and inf. VPs were enriched compared to non-inf. VPs. No significant impact on the oncolytic potency was observed [154]. A TEM analysis showed native bullet-shaped particles as well as round-shaped particles, which has also been reported elsewhere as a processing outcome for VSV particles [83].

The continuous application of membrane adsorbers in a periodic countercurrent chromatography (PCC) setup efficiently uses high flow rates due to low column backpressure while overcoming the limitations of low binding capacity. Due to convective channels, the mass transfer is high, and binding kinetics are relatively independent of flow rates [244]. In a PCC setup, the FT and/or the wash of one column is fed into the next column, achieving a higher loading density on the first column, while unbound VPs bind on the next column. Periodic change of columns achieves a continuous, near-maximal loading and elution of highly concentrated VPs while reducing the required membrane area. The outcome is an increased productivity per membrane area as well as a robustness increase. Matos et al. showcased the application of PCC for the purification of a defective herpesvirus using four membrane units [245]. The challenge in this purification step, namely the low binding strength of VPs and, thus, virus loss during washing was overcome by the post-wash load of the PCC application. A constant yield of over 80% was achieved compared to the variable recovery of 65 to 80% in the batch process [245]. Fortuna et al. applied a pseudo-affinity PCC consisting of 3 membrane units for the purification of inactivated influenza particles and reached a 2.3-fold increase in productivity compared to batch due to efficient utilization of binding capacity [246]. Both examples dealt with the purification of replication-incompetent env. VPs. Applications for replication-competent have only been reported for non-membrane chromatographic systems and non-env. VP [247,248]. It remains to be shown if the continuous PCC approach is also beneficial for replication-competent env. VPs that are more labile and sensitive to environmental stress. PCC systems incorporate a complex fluid stream with more pumps, valves, and flow rate changes, inducing an increased amount of shear stress on particles. Furthermore, PAT sensors in the ATMP field are lacking but are required for dynamic control of PCC and, thus, robust application [245]. While current development is still early, continuous membrane-based PCC systems have the potential to reduce manufacturing costs at a large scale [245].

#### 7.2.3. Monolith Resins

Monoliths share similar properties and advantages over bead-based resins as the membranes discussed earlier. Monoliths are also comprised of convective channels, eliminating diffusional limitations and ensuring a high mass transfer and low backpressure. The difference lies in the polymerization of the resin, which, in the case of the monolith, occurs directly in its final shape—most commonly a hollow cylinder. Thus, no packing or stacking irregularities are observed in monoliths [240,249]. The scalability of monoliths is made possible through the availability of distinct column sizes over a large range of volumes. It is worth noting that monoliths of different base materials exist. In the case of env. VP purification, usually polymeric-based monoliths comprised of channel sizes in the micrometer range are utilized. Silica-based monoliths are available as well; however, they are comprised of additional diffusional pores, which are inaccessible for VPs and are, thus, not suitable [250].

Due to the convective channels, high mass transfer, and low backpressures, monoliths can be loaded with high flow rates without compromising on DBC, as shown for NDV [19] and influenza VPs [202]. However, the binding kinetic itself might be flow rate limiting, as Gerster et al. reported for the purification of BV particles [163]. This limitation is independent of resin type and has also been reported for membrane adsorbers [207,251]. For column screenings at a small scale and analytics, axial flow disks are utilized, which can be stacked in an appropriate housing to achieve a flexible column volume. Dispersion effects have been observed due to a certain heterogeneity of pore sizes, leading to flow variances within the resin, thus reducing resolution [252]. A radial flow design is used for larger scales. To control the polymerization temperature and, thus, channel sizes of monoliths, the resin geometry is limited in its thickness. Thus, a tubular design with radial flow is used for lab-scale as well as production-scale applications [253]. Due to the required flow distribution for radial flow, the void volume in the device is increased, leading to an additional dispersion factor [254].

Monolith resins are available with different channel sizes, which influences the separation performance and flow properties. Larger channels reduce backpressure, and thus, a higher flow rate can be used, which is beneficial for high-volumetric feeds. However, larger channels also reduce the binding capacity due to a less available surface area. Small channels might not be suitable for the purification of large env. VPs, even though the particle size is smaller than the channel diameter. This was observed for mumps VPs using an immunoaffinity monolith [255], mumps and measles VPs using a HIC monolith [45], and NDV on an AEX monolith [19]. In all cases, no flowthrough and low elution of particles were observed for the lower channel size (1.3 µm), whereas larger channel sizes (2 µm or 6 µm) achieved sufficient recovery of inf. VPs. Sviben et al. assumed convective entrapment or increased shear stress for smaller channels to be the cause [45].

Convective entrapment has been shown to reduce recoveries of various VPs as well plasmids in monoliths for small as well as for larger channels [256,257,258,259]. The flow-rate-dependent entrapment in narrowing funnels might be facilitated due to the alternation of narrow and large channel sizes, a structure leading to velocity heterogeneity within the monolith [260]. High flow rates [256,259] and small channel sizes [257] promote entrapment. Low flow rates are impractical in the capture step for VPs where usually a high volumetric feed must be processed. Thus, some extent of entrapment cannot be prevented but should be considered in designing CIP steps to prevent carryover and fouling if monoliths are reused.

Fouling of channels and, ultimately, channel blocking, can also occur for feed material containing high amounts of lipids, as shown for an OH monolith by Burden et al. using a feed derived from a yeast culture [261]. Lipid molecules also compete for binding sites and thus reduce binding capacity if not depleted beforehand. Gerster et al. describe a presumed lipid fouling of an AEX monolith resin leading to reduced recoveries of BV particles [163]. Negatively charged phospholipid fragments become tightly bound to the ligands and capture VPs through membrane fusion. The strong interaction leads to an irreversible capture and reduces recoveries as well as binding capacity. The utilization of an epoxy pre-column column to deplete phospholipid fragments improved the performance of the subsequent AEX monolith (97% VP recovery in all elution fractions compared to 77.6% without a pre-column). An AEX-based purification method for NDV particles was developed by Rogerson et al. [19], showing a factor of 5 to 65 higher DBC compared to bead-based resins. Interestingly, the breakthrough signal surpassed the feed signal in the chromatogram, presumably due to the displacement of retained particles when the monolith is overloaded.

#### 7.2.4. Monolith-like Particles (MLP)

A novel class of bead-based resins are cellulose-based monolith-like particles that comprise large internal pore structures accessible to large biomolecules. Kadoi et al. polymerized spherical beads of approx. 90 µm in an oil/water emulsion having internal pores of 3 µm mode size [262]. The authors modified the beads for mechanical stability and functionalized them using dextran sulfate as a pseudo-affinity ligand. For inactivated influenza particles, these MLPs showed an HA activity recovery rate of 69% compared to commercially available bead-based pseudo-affinity resins with 35% and lower. Impurity reduction levels were comparable across all resins, while determined DBC was highest for the MLP resin. The handling and scalability of MLPs are comparable to conventional bead-based columns, while the functional properties are more like monoliths. Kadoi et al. showed low backpressure while using high flow rates; however, the question of flow-rate-dependent DBC was not addressed. Large particles are slow in diffusion, thus long diffusional paths into beads require long residence times of particles in the column. Furthermore, the suitability of the MLP approach was shown only for inactivated particles up to 100 nm [262].

#### 7.2.5. Cryogels

Cryogels, structurally akin to monoliths, are characterized by a flexible and sponge-like structure. Their manufacturing process, known as cryopolymerization, leverages a frozen phase to instill porosity within the resin [263]. Compared to monoliths, cryogels consist of significantly wider convective channels, ensuring even lower backpressures but also reduced capacities. Additionally, their elasticity can lead to compression of the structure at higher flow rates and, thus, a sudden increase in backpressure [264]. Despite this, cryogels might allow the direct purification of cell cultures without the need for clarification [264]. In addition, the use of acrylamide monomers for polymerization enables inherent hydrophilicity of the resin surface and thus prevents unspecific adsorption of proteins.

The use of cryogels for affinity chromatography of LV particles was previously described; however, difficulties were reported [264,265,266]. A limitation of cryogels is their low binding capacity due to the wide channels. Compared to a monolith, cryogels were shown to exhibit an overall lower performance [265]. Streptavidin-coated cryogels were successfully used to bind biotinylated LVs; however, the particles cannot be eluted [266]. Grafted polymer layers have been added to improve the binding capacities of cryogels, and beneficial use could be shown for protein purification [267]; however, published applications for VPs could not be found.

The application of cryogels might be beneficial if non-clarified harvest can be directly purified; however, properties such as capacity, specificity, and mechanical stability to ensure fast processing of high volumetric feeds are a prerequisite.

#### 7.2.6. Fibers

Functionalized fibers assembled as a non-woven fabric membrane have been developed to achieve scalable, convective-driven chromatography devices with fast mass transfers [268]. For instance, cellulose-based nanofiber adsorbers with channels of around 2–3 µm enable the application for large biomolecules such as VPs. Ruscic et al. used AEX-functionalized RC fibers for the purification of LVs and achieved up to 90% recovery of transduction-active particles [269]. No impact of the nanofiber chromatography step on functional as well as structural viral integrity was observed as evaluated by FT runs and extensive analytical assays such as TEM. The high concentration factor and the possibility to use high flow rates (100 CV/min) rendered it an ideal method for the initial high volumetric reduction step. A 2-log hcP reduction was reached, while hcDNA could not be reduced without nuclease treatment [269]. Using cellulose-based fibers as well, Turnbull et al. evaluated the influence of AEX ligand density and residence time on the infectious recovery of adenoviruses [227]. As discussed in Section 7.1.5, a negative impact of high ligand densities and, thus, reduced recoveries was observed.

#### 7.2.7. Restricted-Access-Media (RAM)

RAM are bead-based resins that feature a functionalized core shielded by a porous layer, thus restricting access to large particles. It is used as a polishing step in FT mode for the removal of small impurities that diffuse through the pores and bind to the core while VPs flow through the resin [270]. RAM can be used irrespective of differences introduced in the genotype of viruses as long as unspecific binding is not introduced, as shown for two orf virus subtypes [70].

Various materials, shell pore sizes, and ligand functionalities are available, 12 of which were challenged by Lothert et al. with clarified harvest containing high impurity levels for an orf purification screening [161]. The authors showed generally similar behavior for the columns, with infectious recoveries in the range of 42% to 87%. Some columns showed a higher capacity for impurity removal; however, these were columns at the lower end of inf. VP recoveries. The choice of RAM depends largely on the feed composition. Shear stress, on-column inactivation, or accessibility of HIC ligands were discussed but not further investigated. Overall, all columns showed low reusability, as impurity reduction worsened after each subsequent cycle, even though thorough CIP steps were applied. In two other studies, Lothert et al. used the CaptoCore700 (CC700, Cytiva, Marlborough, MT, USA) for polishing of orf virus and achieved inf. VP step recoveries of 90% and over 95% [70,271]. The CC700 outperformed other resin and ligand types in terms of recovery and hcDNA depletion [271], while viral integrity remained unimpaired [70].

The application of CC700 as the first chromatographic step before concentration, contrary to its intended use, is reported by Steppert et al. for the purification of measles VPs [67]. The authors loaded clarified harvest directly on the CC700 and yielded an inf. VP recovery of 61%, which was higher than compared to B&E-mode resins. High impurity binding capacities of the CC700 were observed. A scale-up for 8.9 L clarified and nuclease-treated harvest material was processed on a 190 mL column with 4 min column residence time and resulted in near complete inf. VP recovery. However, RAM columns dilute the load instead of concentrating, so a subsequent UF/DF-step for concentration was applied [67].

### 7.3. Chromatography Summary

Conventional chromatography concepts, such as IEX and SEC, have been successfully applied for env. VPs, and new methods such as SXC are being developed and tested for further process improvements. Especially with affinity chromatography, more development can be expected as the field of viral therapeutics continuous to grow and establishes itself amongst biopharmaceuticals. Intelligent ligand design and novel surface functionalization will improve and better modulate binding strength, and more research will be required to fully understand such binding mechanics.

Additional to the conventional chromatographic stationary phases, novel stationary phases are being developed. These are tailored to accommodate large VPs and high volumetric process streams. Overall, convective-driven stationary phases have prevailing advantages due to the higher flow rate limits and increased mass transfer rates.

As different viruses most likely differ in key properties influencing binding behavior and process knowledge is unfortunately limited, screening experiments are essential to ascertain the optimal chromatography modalities for each process step.

## 8. Further VP Purification Techniques

### 8.1. Flocculation

Alternative purification methods have been explored or transferred from other fields for the purification of env. VPs in occasional studies. For instance, flocculation is conventionally used for viral clearance, but promising results for purification applications have been shown as well [272]. By the addition of high salt or osmolyte concentrations, VPs are specifically flocculated, meaning aggregated. This leads to an even bigger size difference of VPs compared to non-flocculated impurities, and the flocculated VPs are retained in a subsequent filtration step. Diluting the filter retentate afterward reduces the flocculation agent concentration and reverses the flocculation, thereby recovering inf. VPs. This concept was used for enveloped Sindbis VPs, and realized as a three-step filtration process with the initial purification step achieving recoveries of up to 96% [273]. Removal of large amounts of protein was reported, but hcDNA co-aggregated with VPs and, thus, was co-purified. The authors utilized mannitol as a flocculation agent which was shown to work also for a non-enveloped virus, although with a lower recovery rate [273]. Hasan et al. reviewed the utilization of osmolytes as excipients and flocculation agents for vaccine VPs [274]. The authors highlighted the stabilizing effects of osmolytes, contrary to high salt concentrations, and their use as adjuvants and concluded there might not be any need for removing these agents after flocculation.

### 8.2. Aqueous Two-Phase (ATP) System

Aqueous two-phase (ATP) systems utilize two immiscible aqueous phases, each characterized by distinct partition coefficients for VPs, hcDNA, and hcPs. This differential partitioning facilitates the effective separation of target molecules from impurities. One of the key features of ATP systems is the use of polymers, which can also aid in stabilizing env. VPs. The gentle processing method avoids harsh conditions and minimizes unwanted interactions with the VPs [275]. Various applications for enveloped VLPs or inactivated env. VPs show general good recoveries and purification performances. The flexibility of this system was shown in a continuous setup with the use of an in-line helical mixer and separator, reaching nearly 100% recovery [276]. Optimization possibilities were shown with different polymer chain lengths, e.g., PEG size [277] and osmolyte addition [278]. The only reported application for the purification of infectious VPs was published by Kim et al. for Hepatitis C VPs [279]. A PEG/Dextran system and subsequent low-speed centrifugation for VP recovery was applied, yielding in an inf. VP recovery of 40%.

## 9. Formulation and Storage

For a safe and effective drug product, it is necessary to maintain high inf. VP titers throughout long-term storage. Storage of VPs is typically in a dried state or at ultra-low temperature. Functional integrity must be preserved through a stable structural integrity, and causes of particle degradation must be eliminated. Physical and chemical factors resulting in the degradation and loss of particles include freeze/thaw damage, pH changes, surface adsorption, shear stress, oxidative denaturation, and thermal stress. Pan et al. provide an extensive evaluation of these factors and describe formulation strategies for VPs for preventing or at least minimizing their negative impacts [280]. Membrane proteins, exposed on the outside of env. VPs, are especially prone to degradation. Surface glycoproteins such as spike proteins enable attachment to host cells and thus initiate the viral replication cycle. Their functionality must be preserved, and the task of VP formulation is thus similar to protein formulation.

At the end of a production process, env. VPs, and also other VPs, undergo buffer exchange into the final formulation buffer. Direct addition of excipients may also be required prior to a sterile filtration step. The formulation buffer composition depends on the biophysical properties of the VPs, along with the desired storage conditions. In addition, safe patient administration must be achieved. Although the mechanisms of storage degradation processes and their prevention by excipients are understood to some extent [280], a thorough screening of stabilizing agents is usually needed [281,282]. For each VP in development, forced degradation studies are performed to define the space for later formulation, including FT-cycle stability, pH and salt tolerability, temperature and mechanical stability, and propensity for aggregation with test excipients [281,282]. Beyond that, formulation screenings involve stability studies at storage conditions and accelerated storage conditions [281,282]. For instance, Homan et al. reviewed stability studies of env. and non-env. VPs in liquid formulations which were stored at different temperatures [283]. They found that lower temperatures correlated with longer functional stability. It is noteworthy that the authors pointed out the importance of storing VPs below the solution’s glass transition temperature (Tg), at which molecules’ and atoms’ severely limited motion prevent degradation processes. Tg is estimated for VP solutions to be between −35 °C and −60 °C. At temperatures of at least 50 °C below Tg, reached only in liquid nitrogen tanks, almost infinite stability is reached. Homan et al. had access to stability studies of seven different virus strains and developed a generalized stability model, predicting long-term recovery losses. In these long-term stability studies, degradation due to the freezing and thawing process is excluded from the results and evaluated in specialized studies. An extrapolation over 10, 20, and 30 years of VP solution stored at −70 °C predicted infectivity losses to be lower than 0.3, 0.5, and 0.7 log_10_, respectively [283].

### 9.1. Liquid Formulation

VPs stored in a liquid formulation for drug products require aliquoting into small-volume containers and the subsequent freezing and storage at temperatures below −70 °C [283]. The formulation is designed to maintain VP stability during the FT-cycle, and also while in frozen storage. Various buffer components and excipients can be used to maintain particle infectivity during this process.

Buffering agents are essential in maintaining the pH of the formulation, which is critical for the stability and activity of the VPs. Common buffering agents include phosphate buffers, histidine, and citrate. These agents stabilize the pH at a virus-specific optimal setpoint, thus preventing pH-induced degradation. During freezing, the pH of the buffer solution can change drastically due to the successive crystallization of buffer substances. The intensity of this change depends on the type and concentration of the buffer components. Salts can be added to mitigate pH shift, and amorphous excipients like sucrose or alginate can inhibit buffer crystallization [280].

The concentration changes upon freezing can also result in osmotic pressure impacting the viral integrity as well as the VP’s surrounding hydration layer, which influences membrane protein functionality. Virus inactivation, degradation, and aggregation follow. Cryoprotectants serve as stabilizing agents, reducing ice crystal formation, balancing the osmotic pressure, and replacing water molecules bound to proteins. Cryoprotectants used in viral formulations include saccharides (e.g., sucrose, trehalose), polyols (e.g., sorbitol, PEG), and albumin, as well as cations such as calcium and magnesium [280,282].

Surface adsorption of VPs results in particle aggregation and is accelerated through mechanical forces like stirring and shaking. Surfactants such as polysorbate and poloxamer are used to prevent adsorption through the shielding of interfaces [284]. However, polysorbate degradation by residual enzymatic hcPs is a known challenge in industry [285] and degradation products of polysorbate were shown to inactivate env. VPs [286]. Oxidation processes can alter membrane protein and lipids of env. VPs resulting in inactivation [178,280]. Cellular proteins such as albumin, as well as amino acids such as methionine and histidine, were shown to protect VPs from oxidation.

### 9.2. Lyophilized Formulation

The subsequent sublimation process, the removal of water from virus formulations, further stabilizes virus preparations. After lyophilization, the dried preparations can be stored at higher temperatures, for live vaccines usually at −20 °C or, as reported by Hansen et al., between 2 and 8 °C [287]. This reduces the requirements on storage and logistics but, at the same time, introduces another process step, potentially reducing the infectivity of VP. Lyophilized formulations require the addition of a bulking agent to guarantee the formation of a solid cake when lyophilized. Commonly used bulking agents include mannitol and amino acids such as glycine [280]. Env. VPs, as highly sensitive particles, require a high amount of stabilizers and complex formulations to facilitate sufficient inf. VP recovery [288,289]. These formulations necessitate a high development effort. An alternative to lyophilization is spray drying, which embeds the VPs in a glassy matrix of, e.g., starch, thus preserving its function [289]. The potential detrimental freezing process is prevented, and the water is evaporated. Coleman et al. compared both processes for the storage of BV. No loss of infectivity was observed for spray drying or lyophilization. Spray-dried VPs showed enhanced stability against high temperatures of, e.g., 85 °C for 1 h or 30 °C for one week, which significantly reduces storage requirements [289].

## 10. Summary and Conclusions

Process development for replication-competent env. VPs for therapeutic purposes is particularly challenging due to their high sensitivity to environmental stress. The functional integrity of these particles is crucial for a safe and effective treatment, but the manufacturing process imposes a negative impact and challenges this integrity. Thus, thorough evaluation and then optimization of potential impacting parameters are indispensable.

Advanced analytical methods are essential to assess the impact of process steps on particles and determine their quality and integrity. A combination of such methods is required to evaluate VP preparations completely, and methods include infectious titer assays, total virus particle quantification, and structural analysis.

A comprehensive overview of the current state of bioprocessing for replication-competent env. VPs is provided in this review. Bench-scale centrifugation methods for concentration and purification are less effective compared to contemporary chromatographic techniques, which offer higher recovery and purity. Both conventional and modern chromatographic techniques are evaluated, with variable reported recoveries across different publications, mainly dependent on the viruses studied. Table 2 presents an exemplary selection of contemporary and scalable purification processes for several env. VPs. Differences in purification strategies due to specific VP properties and virus life cycles are apparent. For intracellular VPs, cell disruption is necessary, but it introduces additional impurities that burden downstream processes. The orf process described by Lothert et al. utilizes an FT-cycle for cell disruption and requires a subsequent filtration train for clarification, with a coarse primary filtration step [70]. In the measles process by Steppert et al., a single and coarse clarification filter is sufficient due to the subsequent RAM chromatography step in FT mode [67]. This enables a gentle initial processing for the sensitive measles VPs, however requires a subsequent UF/DF step for concentration. Upstream conditions significantly influence VP quality and heterogeneity, with continuous harvest concepts aiming to mitigate the adverse effects of the cell culture environment. However, Gränicher et al. compared the batch and continuous harvest bioprocessing of vaccinia VPs, showing similar results in overall yield and impurities [129]. However, the final VP titers are not explicitly stated. Nuclease treatment is often necessary to degrade hcDNA and improve the processability of the virus feed. This is also observed in Table 2, in which the sole use of fine filters (≤1 µm) for clarification is only applied if the nuclease step is applied prior to clarification. Filtration methods are ubiquitously used in the purification process of env. VPs, but careful selection of filter materials and optimization of pore sizes are necessary to maintain viral integrity and achieve high yield. Various UF/DF modalities and filter materials are applied in the referenced processes in Table 2, each optimized to the specific VP properties. Convective-driven stationary phases are beneficial for processing high volumes without mass transfer resistances, especially immediately after the harvest stage. This is also reflected in Table 2 with the use of solely membrane stationary phases for B&E chromatography steps.

Emerging trends and technologies in the field of VP purification include the development of more specific and high-throughput analytical methods, continuous processing techniques, and advanced chromatographic methods. These advancements have the potential to significantly impact the field by improving the efficiency and robustness of VP purification processes. However, every virus is different, and purification approaches need to be tested for every virus, especially if the separation principle relies on virus-specific characteristics such as size, structure, stability, and surface proteins. Many approaches have proven beneficial for vaccine or virus-like particle (VLP) applications, but method transfer and optimization for more sensitive replication-competent env. VPs are often still outstanding.

This review highlights the demand for optimized purification strategies to ensure the safety and efficacy of virus-based therapeutics. By addressing the complexities and providing insights into best practices, valuable knowledge is contributed to the field of viral bioprocessing, ultimately supporting the development of effective and stable viral therapies and vaccines.

## Figures and Tables

**Table 1:** Selected DNA and RNA enveloped virus species currently in development as oncolytic virus or cancer vaccine.

	Virus Family	Particle Geometry	Genome Size	References
**DNA viruses**				
Herpes simplex virus (e.g., HSV-1)	Herpesviridae	155–240 nm, icosahedral	152 kb	[14]
Orf	Poxviridae	220–300 × 140–200 nm, ovoid shaped	140 kb	[15]
Vaccinia	Poxviridae	360 × 270 × 250 nm, brick shaped	190 kb	[14]
Myxoma	Poxviridae	320 × 235 nm, brick shaped	162 kb	[16,17]
**RNA viruses**				
Measle	Paramyxoviridae	100–300 nm, helical	15.8 kb	[14]
VSV (vesicular stomatitis virus)	Rhabdoviridae	70 × 200 nm, bullet shaped	11 kb	[14,18]
NDV (Newcastle disease virus)	Paramyxoviridae	100–500 nm, spherical	15 kb	[14,19]
LCMV (lymphocytic choriomeningitis virus)	Arenaviridae	78–90 nm, spherical	10.6 kb (segmented)	[20,21,22]
Influenza	Orthomyxoviridae	50–120 nm, spherical + longer filamentous forms	13.6 kb	[23,24]

**Table 2:** Selected examples include technical details of scalable VP purification processes of VPs within the scope of the review (replication-competent, enveloped VPs with therapeutic application potential). No complete process descriptions including infectivity analysis could be found for myxoma virus, NDV, LCMV, and influenza. Abbreviations: adh.—adherent; susp.—suspension; conc.—concentration; n.a.—not applicable. Manufacturer information: c-LEcta (Leipzig, Germany), Cytiva (Marlborough, MT, USA), Merck (Darmstadt, Germany), Sartorius Stedim (Goettingen, Germany), Spectrum Laboratories (Rancho Dominguez, CA, USA).

Virus	Upstream		Downstream					**Final Product**	**Process Performance**
	Cell Culture	Virus Harvest and Release	Nucleic Acid Digestion	Clarification (Primary and Secondary)	1st Purification (Capture)	2nd Purification (Polish)	**Sterile Filtration**	**Infectivity, Dosage, and Impurities**	**IU Overall Process Yield**
HSV-2 [290], (repl.-def. vaccine production)	Adh. Vero cells, MOI 0.01, TOH 24–72 hpi	Dextran sulfate, 100 µg/mL, up to 24 h, further processing of supernatant	e.g., Benzonase (Merck): 90 U/mL + 5 mM MgCl₂, at 25 °C for 4–6 h	Filtration (e.g., Sartopure PP2, 0.65 µm (Sartorius Stedim))	AEX (e.g., Mustang Q membrane (Cytiva)): high-salt elution (2 M NaCl)	UF/DF (e.g., hollow fiber PS, 100 kDa, Spectrum Laboratories): TFF conc. (5–10×), DF (3–5×)	n.a. due to VP size, aseptic process suggested	>1 × 10^7^ pfu/mL hcDNA < 10 ng/dose hcP: 30 µg/mL 10^7^ pfu/dose	10% to 20%
Orf [70]	Adh. Vero cells, MOI 0.05, TOH 120 hpi	Intracellular VP release by a FT-cycle, further processing of complete broth	Benzonase (Merck): 250 U/mL, 1 h at RT, after clarification	Filtration 5 µm and 0.65 µm, Sartopure PP3 (Sartorius) or Millistak cellulose with DE (Merck)	SXC binding at 8% PEG8000 on RC membrane stack (pore size 1 μm)	RAM: CC700 (Cytiva)	not discussed	1.1–4.2 × 10^6^ IU/mL total DNA: ~1 ng/dose total protein < LOD 10^6^ IU/dose	64%
Vaccinia [129], (MVA, batch process)	Avian susp. cells, MOI: 0.05, TOH at cell viability ~70%	Cell culture broth was further processed	Denerase (c-LEcta): 35 U/mL + 3 mM MgCl₂, 4 h 37 °C, after clarification	Primary: Acoustic settler (3 W, 2.1 MHz, 252 mL/h) Secondary: Filtration 0.45 µm, Sartopure PP3 (Sartorius Stedim)	Pre-filtration (0.45 µm, CA SXC: binding at 7.2% PEG6000 on RC membrane stack (pore size 1 µm)	n.a.	not discussed	>5 × 10^7^ TCID_50_/mL hcDNA < 10 ng/dose total protein: 11–37 μg/dose 1.4 × 10^8^ TCID_50_/dose	55%
Vaccinia [129], (MVA, continuous perfusion process)	Avian susp. cells, MOI 0.05, TOH start 40 hpi	Continuous harvest of cell culture broth	Denerase (c-LEcta): 37 U/mL + 4 mM MgCl₂, 4 h 37 °C, after clarification	As batch process	As batch process	n.a.	not discussed	Titers as batch process hcDNA < 10 ng/dose total protein: ~10 μg/dose 1.4 × 10^8^ TCID_50_/dose	51%
Measles [67]	Adh. Vero cells, MOI: 0.001 to 0.01	Supernatant was further processed	Benzonase (Merck): 50 U/mL + 2 mM MgCl_2_ for 1 h 37 °C after clarification	Filtration, 3 µm: Sartopure PP3 (Sartorius Stedim)	RAM: CC700 (Cytiva)	UF/DF (cellulose flat sheet membrane, 100 kDa, Merck): TFF conc. (~9×), DF (5×)	n.a. due to VP size, aseptic process suggested	7.9 × 10^6^ TCID_50_/mL dsDNA: 354 ng/mL → 18 ng/dose hcP: 18 µg/mL → 1 µg/dose 10^5^ IU/dose	only step yields are disclosed, see reference
VSV [154], (rVSV-NDV, fusogenic virus)	Avian susp. cells, MOI 0.0001, TOH at cell viability ~90% (between 48 and 67 hpi)	Cell culture broth was further processed	Denerase (c-LEcta): 20 U/mL + 2 mM MgCl_2_, 1 h at RT	Primary: Filtration 1–0.4 µm Millistak CE (Cytiva) Secondary: Filtration 0.45 µm Sartopure PP3 (Sartorius Stedim)	AEX membrane Sartobind Q (Sartorius): Elution at 1.2 M NaCl	Pre-dilution (4×), UF/DF (PES hollow fiber, 750 kDa, Cytiva): TFF conc. (4×), DF (6×)	Supor EKV Mini Kleenpak filter 0.2 µm (Cytiva)	4 × 10^9^ TCID_50_/mL total DNA: 1.4 ng/mL total protein: 0.3 µg/mL 5 × 10^7^ to 5 × 10^10^ IU/dose	65%

## Data Availability

No new data were created or analyzed in this study. Data sharing is not applicable to this article.

## Abbreviations

The following abbreviations are used in this manuscript:

AAV	adeno-associated viral vectors
AEX	anion-exchange chromatography
ATF	alternating TFF
ATMP	advanced therapy medicinal product
ATP	aqueous two-phase
B&E	bind-and-elute
BV	baculovirus
CA	cellulose acetate
CC700	CaptoCore700
CEX	cation-exchange chromatography
CIP	cleaning-in-place
DBC	dynamic binding capacity
DE	diatomaceous earth
DENV	dengue virus
DF	diafiltration
DIP	defective interfering particle
DLS	dynamic light scattering
DNA	deoxyribonucleic acid
ELISA	enzyme-linked immunosorbent assay
EM	electron microscopy
EU	European Union
EV	extracellular vesicle
FDA	food and drug administration
FFF	field flow fractionation
FMDV	foot-and-mouth-disease virus
FT	flowthrough
FT-cycle	freeze/thaw cycle
GF	glass fibers
HA	hemagglutination assay
HIC	hydrophobic interaction chromatography
HIV	human immunodeficiency viruses
HPLC	high-performance liquid chromatography
HPV	human papillomavirus
HSV	herpes simplex virus
IEP	isoelectric point
IEX	ion exchange chromatography
IU	infectious unit
LCMV	lymphocytic choriomeningitis
LS	light scattering
LV	lentivirus
MALDI-MS	matrix-assisted laser desorption/ionization MS
MALS	multi-angle LS
MLP	monolith-like particles
MOI	multiplicity of infection
MS	mass spectrometry
MVA	modified vaccinia ankara
NC	nitrocellulose
NDV	Newcastle disease virus
NFF	normal flow filtration
NGS	next-generation sequencing
NTA	nanoparticle tracking analysis
NYL	nylon
OV	oncolytic virus
P:IU	particles-to-infectious unit ratio
PAT	process analytical technology
PCC	periodic countercurrent chromatography
PCR	polymerase chain reaction
PEG	polyethylene glycol
PES	polyethersulfone
PFAS	polyfluoroalkyl substance
PP	polypropylene
PS	polysulfone
PVDF	polyvinylidene fluoride
RAM	restricted access media
RC	regenerated cellulose
RI	refractive index
RID	radial immunodiffusion
RNA	ribonucleic acid
RSV	respiratory syncytial virus
SBC	static binding capacity
SC	sucrose cushion
SEC	size exclusion chromatography
SLS	static LS
SMB	simulated moving bed
STR	stirred tank reactor
SXC	steric exclusion chromatography
T-VEC	Talimogene laherparepvec
TCID50	50% tissue culture infective dose assay
TEM	transmission electron microscopy
TFDF	tangential flow depth filtration
TFF	tangential flow filtration
TMP	transmembrane pressure
TOH	timepoints of harvest
TOI	timepoint of infection
UF	ultrafiltration
UV	ultraviolet
VLP	virus-like particle
VP	virus particle
VSV	vesicular stomatitis virus
aMPV	avian metapneumovirus
cGE	capillary gel electrophoresis
cyro-EM	cryogenic EM
dPCR	digital PCR
ddPCR	digital droplet PCR
env. VP	enveloped VP
hcDNA	host cell DNA
hcP	host cell protein
hpi	hours post-infection
inf. VP	infectious VP
non-env. VP	non-enveloped VP
non-inf. VP	non-infectious VP
nsEM	negative staining EM
pfu	plaque-forming units
qPCR	quantitative PCR
